# Spontaneous Mutations Occur More in Highly Transcribed Regions in *Daphnia*

**DOI:** 10.1093/gbe/evag021

**Published:** 2026-01-27

**Authors:** Jeremy E Coate, Eddie K H Ho, Sarah Schaack

**Affiliations:** Department of Biology, Reed College, Portland, OR 97202, USA; Department of Biology, Reed College, Portland, OR 97202, USA; Department of Biology, Reed College, Portland, OR 97202, USA

**Keywords:** transcription-associated mutagenesis (TAM), transcription-coupled repair (TCR), base substitutions, insertions, deletions, Cladocera

## Abstract

Many molecular processes (eg replication, recombination, and transcription) use DNA as a template molecule, which may lead to an increase or decrease in the likelihood of spontaneous mutation and/or repair of mutations to this key information storage molecule. In the case of transcription, both positive and negative correlations with the likelihood of mutation have been observed across species, which have formed the basis of two proposed mechanistic models: transcription-associated mutagenesis and transcription-coupled repair. Here, we examine the patterns of spontaneous mutations in regions of low and high transcription in two species of the aquatic microcrustacean, *Daphnia*. By mapping events from a long-term mutation accumulation study (*n* = 66 lineages derived from nine different genotypes from three populations) with multiple, large-scale publicly available RNA-seq datasets, we find that mutations are more frequently observed in regions of high transcription in *D. magna*, as well as in the congener, *D. pulex*. The results are robust across mutation types (base substitutions, insertions, and deletions) and among transcriptional profiles (across developmental stages and environmental conditions). Overall, the positive correlation was robust to different methodological approaches and when controlling for other genomic features (like GC-content). Based on our observations, transcription-associated mutagenesis provides a more likely explanation for the positive relationship between mutation accumulation and transcription levels observed in *Daphnia*. Characterizing such patterns is important for understanding the evolution of genes, differentially expressed regions of the genome, and the mutation rate.

Significance Although many molecular processes use the same template molecule (including DNA replication, recombination, repair, chromatin remodeling, and transcription), the correlation between these processes and the likelihood of mutation has rarely been examined outside classic model systems. Here, we examine the net relationship between transcription and mutation, for which models of both positive (transcription-associated mutagenesis) and negative associations (transcription-coupled repair) have been proposed. While past studies have yielded contradictory results, in *Daphnia*, there is a clear, consistent, and positive correlation between local expression levels and the frequency of mutation. This finding has significance not only for the evolution of highly expressed regions of the genome and the likelihood of mutation-induced disease but also for the evolution of mutation rates across genomes and environments.

## Introduction

Many molecular processes rely on the same template molecule; for example, the replication, recombination, repair, and transcription of DNA. A longstanding question in molecular evolution is whether any of these processes, individually or in combination, have a positive or negative effect on local mutation rates (reviewed in Finkelstein and Greene 2013). Transcription, in particular, is of interest, as it has long been argued that it could both increase or decrease the likelihood of mutation via two main routes: transcription-associated mutagenesis (TAM) and transcription-coupled repair (TCR; Kim and Jinks-Robertson 2012). If transcription renders the non-template single-strand DNA more vulnerable to mutations, as proposed in the TAM model, there should be more mutations observed in regions of higher transcription (reviewed in Jinks-Robertson and Bhagwat 2014). In contrast, if RNA polymerase stalls at damaged sites on the template strand, it could pave the way for more effective DNA repair as models of TCR predict (reviewed in Hanawalt and Spivak 2008; Spivak 2016; Duan et al. 2021). These two scenarios are not mutually exclusive, however, and both mechanisms have been observed in a wide range of taxa (Martincorena et al. 2012; Park et al. 2012; Martincorena and Luscombe 2013; Krasovec et al. 2017; Strick and Portman 2019; Liu and Zhang 2020; Xia et al. 2020). Despite these investigations, whether the net effect of transcription on the likelihood of mutation is positive or negative, in general, remains unknown.

### Transcription-Associated Mutagenesis (TAM)

Evidence for TAM was first shown in bacteria by Herman and Dworkin (1971) and Jinks-Robertson and Bhagwat (2014). The non-transcribed strand is exposed both within the transcription bubble and, in eukaryotes, in structures known as R-loops that can form downstream (Santos-Pereira and Aguilera 2015). Transcription has also been shown to be mutagenic on the transcribed strand (Hendriks et al. 2010). Supercoiling near transcription bubbles and the associated torsional stress, as well as its resolution by topoisomerases, can be mutagenic as well (Lippert et al. 2011). Damage to nucleic acids can result from conflicts between transcription and both the replication (Schroeder et al. 2020; Lalonde et al. 2021) and/or repair of DNA (Selby and Sancar 1990).

### Transcription-Coupled Repair (TCR)

The notion that transcription increases the accessibility of DNA to repair machinery, thus lowering local mutation rates, was first posited by Mellon et al. (1987). As a sub-pathway of nucleotide excision repair (NER), TCR can counteract the mutational process when RNA polymerase encounters a damaged base (Spivak 2016; Strick and Portman 2019). This encounter stalls transcription and triggers the recruitment of DNA repair machinery (transcription-coupled NER; Lans et al. 2019; Duan et al. 2021), which preferentially detects and repairs lesions on the transcribed strand, resulting in a strand bias for mutations (Strick and Portman 2019). However, NER is also triggered by global genomic repair, which is independent of transcription, and largely eliminates the TCR-induced mutational strand bias.

### What is the Net Influence of Transcription on the Likelihood of Mutation?

Early studies utilizing reversion assays of inducible genes in bacteria were the first to show a positive correlation between transcription and mutation overall (eg Herman and Dworkin 1971; Savić and Kanazir 1972). Decades later, similar approaches in yeast and human cell lines provided the first evidence for TAM in eukaryotes (Korogodin et al. 1991; Datta and Jinks-Robertson 1995; Bachl et al. 2001; Kim et al. 2007). More recently, Park et al. (2012) used RNA-seq to show that expression levels at mutated sites in yeast mutation accumulation (MA) lines were higher than expected by chance. In addition, using comparative genomic approaches in both yeast (*Saccharomyces cerevisiae* and *S. pombe*) and primates (human and macaque), Park et al. also (2012) showed that intronic substitution rates increased with increasing expression.

But negative correlations have also been observed; for example, Lippert et al. (1998) observed reduced mutation rates with increasing expression using an inducible reporter system in human cells. In plants, Krasovec et al. (2017), using whole-genome sequencing of MA lines in four species of marine algae, observed 2-fold higher mutation rates in intergenic than in genic regions. In addition, within intergenic regions, expression levels were lower at mutated sites than at non-mutated sites, further suggesting that transcription reduces mutation rates. Meanwhile, analyzing MA lines of *Drosophila melanogaster*, Keightley et al. (2009) found that mutations were no more or less likely to occur in genes than expected by chance, suggesting that TAM and TCR may effectively cancel each other out.

In some cases, reanalyses of the same data yield contradictory conclusions. Using the same yeast MA lines, Zhu et al. (2014) found no relationship between mutation and transcription, whereas Chen and Zhang (2014) concluded that mutation rates are elevated in highly transcribed genes. Similarly, Xia et al. (2020) and Xia and Yanai (2022) concluded that widespread transcription in the testes of mouse and human explained TCR-based reductions in mutation (the “transcriptional scanning hypothesis”), but this was refuted by Liu and Zhang (2020), who found the opposite pattern in the same data. These apparent contradictions could be because assessing the net effects of TAM and TCR requires controlling other factors that can correlate with transcription and/or mutation (eg GC-content, nucleosome occupancy, and whether DNA is replicated early or late in S phase; Hwang and Green 2004; Stamatoyannopoulos et al. 2009; Park et al. 2012; Wilson et al. 2015; Gonzalez-Perez et al. 2019; Liu and Zhang 2020). In addition to these challenges, most previous studies are limited to yeast, typically using only one genotype per study (Park et al. 2012; Chen and Zhang 2014; Zhu et al. 2014; but see also Keightley et al. 2009; Krasovec et al. 2017). In many studies, transcription is not measured directly (rather, genic and intergenic regions have been compared) or is limited to transcription levels from a single environmental condition.

Here, we examine the relationship between mutation and transcription in *Daphnia* using sequence data from a long-term MA study with nine genotypes of *D. magna*, multiple publicly available RNA-seq datasets (Giraudo et al. 2017; Russo et al. 2018; Poulsen et al. 2021), and data from a congener for which mutation and transcription data are also available (*D. pulex*). Previously, we have reported highly variable mutation rates across genotypes and among types of mutation (Ho et al. 2019, 2020, 2021; Ho and Schaack 2021). Such high levels of variation in mutation parameters underscore the need for determining proximate, local mechanisms that may generate variation in mutation rates, thus illuminating how this key trait might evolve among lineages and over time.

## Results

**Fig. 1.**
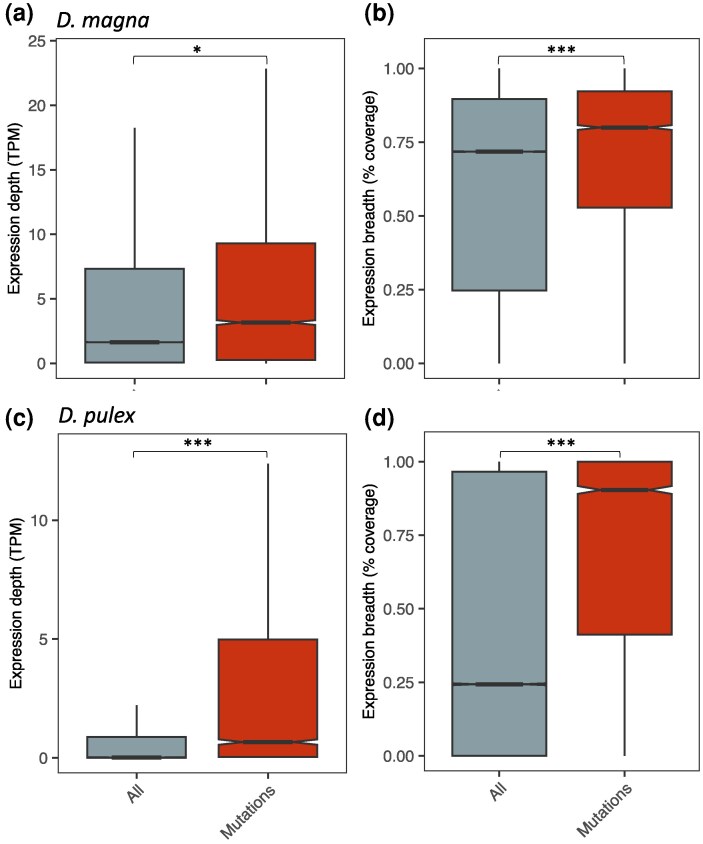
Expression levels are positively correlated with mutation. Median expression depth (transcripts per million [TPM]; left) and breadth (% coverage; right) compared between 10 Kb windows overlapping mutations (mutations; right) compared to those from the full genome (all) in *Daphnia magna* (top) and *D. pulex* (bottom). a) Expression depth, *D. magna*; b) expression breadth, *D. magna*; c) expression depth, *D. pulex*; d) expression breadth, *D. pulex.* For *D. magna*, plots show expression depth or breadth across all 9 genotypes, 3 developmental stages, and 2 conditions. For *D. pulex*, plots show expression depth or breadth for ovaries in a single genotype. Asterisks denote significance: *P < 0.01. ***P < 0.0001.

**Fig. 2.**
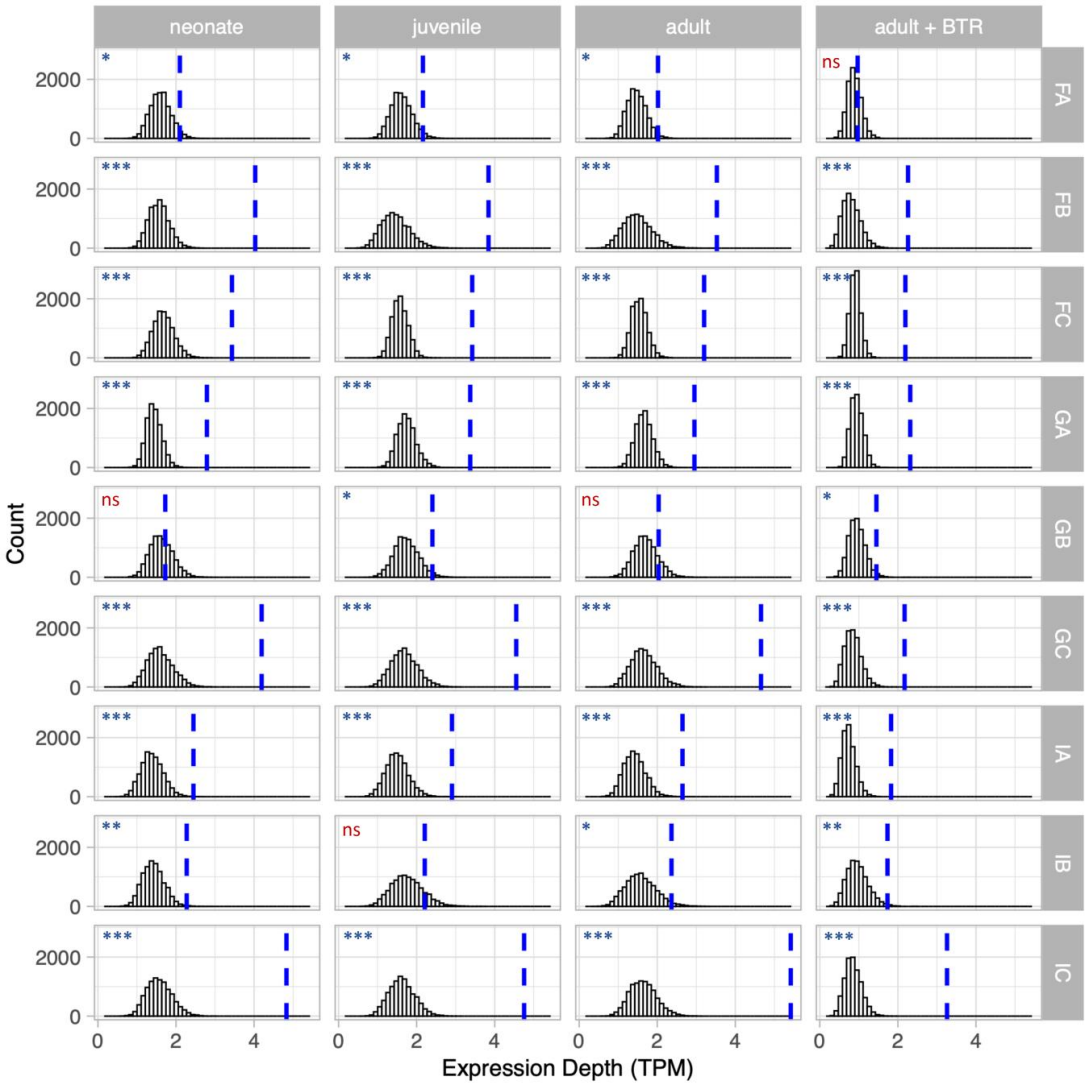
Permutation tests show elevated expression depth in windows with mutations for *D. magna* from nine genotypes, at three developmental stages, and raised in two environmental conditions (adults grown with or without exposure to 1H-benzotriazole). Distribution of median expression depths (transcripts per million [TPM]) from 10,000 permutations of randomly selected windows (black bars) are lower than the median expression breadth for mutation-containing windows (dashed blue line) in 32 of 36 tests performed. Significance values are shown for each genotype-developmental stage-condition combination (*, *P* < 0.05. **, *P* < 0.01. ***, *P* < 0.001. ns, *P* > 0.05).

**Fig. 3.**
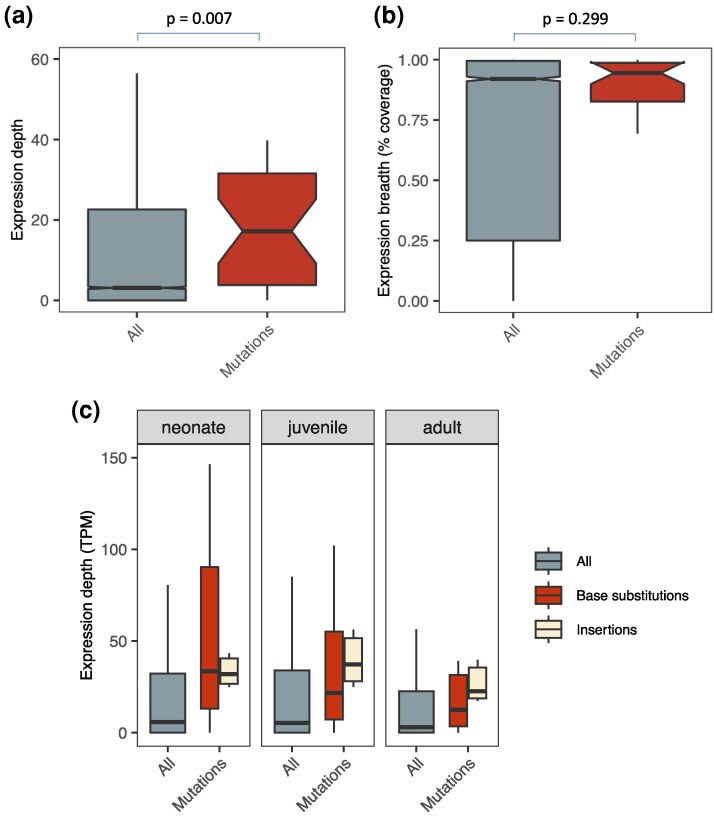
A positive correlation between transcription and mutation even when restricted only to genic regions. a) Expression depth (TPM) for all annotated genes (all) and for genes overlapping mutations (mutations) in *D. magna* (genotype GC). b) Expression breadth (% coverage) for all annotated genes (all) and for genes overlapping mutations (mutations) in *D. magna* genotype GC. c) Expression level for genes that incurred mutations (mutations) versus all genes (all) in the MA line GC, with mutations separated by type (insertions or base substitutions − no deletions occurred in genes).

Transcription is higher in regions of the genome where mutations were found, regardless of whether depth or breadth was used to measure expression levels, in both species of *Daphnia* examined (Fig. 1a; Figs. S1 and S2). Permutation tests reveal this pattern is robust not only across species, but across genotypes, developmental stages, and environmental conditions (Fig. 2; Table S1; Fig. S3), with only 2 of 36 tests not exhibiting a statistically significant difference using either depth or breadth. The result was also observed regardless of window size (Fig. S4) and when only restricted to genic regions (Fig. 3). The fact that *D. pulex* mutations accumulated more in regions of the genome that are highly transcribed in ovaries, specifically, suggests not only that the net mutagenic effect of transcription is characteristic of the genus, but also that the positive correlation exists whether looking at transcription levels in RNA-seq datasets from whole body extractions or germline-specific tissue. When controlling for GC-content (Figs. S5 to S7), the pattern was slightly less strong, but still prevalent. Furthermore, we interrogated the data the opposite way; rather than only asking if windows containing mutations have higher expression, we tested if regions of the genome with high expression contain more windows with mutations, and there was also a positive correlation (Fig. S8). Lastly, the overall pattern (elevated transcription levels in windows containing mutations) did not change when looking at different mutation types separately (base substitution mutations, insertions, and deletions; Fig. S9a and b).

## Discussion

When DNA is in a single-stranded state, as it is during transcription, it may be both more vulnerable to mutation and more available for DNA repair. Two counteracting mechanisms, transcription-associated mutagenesis (TAM) and transcription-coupled repair (TCR), have been proposed, but the net mutagenic effect of transcription on the likelihood of mutation remains elusive. Here, in two species of *Daphnia*, we show that mutations accumulate more in regions of the genome that are highly transcribed, adding to the empirical evidence supporting a positive correlation between high transcription levels and mutation. The pattern is robust across methodologies, genotypes, developmental stages, and environmental conditions. Furthermore, when controlling for GC content and genic/non-genic regions, the pattern persists, suggesting it is driven by transcriptional activity, and not by other genomic characteristics. Finally, although slightly more variable, the pattern is observed generally across base substitutions, insertions, and deletions, suggesting the pattern is robust across a wide array of mutation types, even though high levels of transcription might be predicted to promote or protect against certain types of mutations based on the specific mechanisms involved. Future studies surveying the pattern by looking at microsatellite mutations or transposable element mobilization would be particularly interesting, as the types of mutations are often found to occur most frequently in *Daphnia* (Ho et al. 2019; Ho and Schaack 2021).

It is important to note that the mutations we mapped occurred in the germline, while expression patterns for *D. magna* were based on publicly available whole body RNA-seq datasets that reflect transcription levels largely in somatic cells. The expression data for *D. pulex*, however, were from a dataset using RNA collected from ovaries, which are likely to be at least more germ cell-rich. There is a baseline assumption that the regions that were highly transcribed across libraries experience higher transcription rates in general, but an RNA-seq dataset from germ cells of *D. magna* would be a key direction for future investigation. In addition, a growing number of genomic resources are now available for *Daphnia* (eg Chaturvedi et al. 2023), making it possible to expand the number of expression profiles analyzed and to, perhaps, identify especially mutagenic environmental conditions, either via transcriptional upregulation or other molecular mechanisms. Indeed, some studies taking a fine-grained approach have shown positive correlations between transcription and mutation vary among individuals (Cui et al. 2012), tissues, or even among genes (Cao et al. 2024), consistent with the possibility that this variation is something selection may act upon to shape traits like the mutation rate among lineages over time. Ultimately, the ongoing investigation of the relationship between transcription and genome instability as a component of evolutionary changes to mutation parameters or as a key determinant of the likelihood or consequence of diseases like cancer (Hanawalt 1994; Milano et al. 2024; Bayona-Feliu and Aguilera 2025) requires foundational experiments harnessing the power of experimental evolution and emerging model systems.

## Methods (See Supplemental Methods for Full Details)

In brief, MA lineages and controls were initiated from nine genotypes of *D. magna* from three populations (Finland, Germany, and Israel). The MA lines were propagated by single-progeny descent to minimize selection for an average of 12 generations over ∼2.5 years. Tissue from MA and control lines was used to prepare libraries for paired-end short read whole genome sequencing (∼50 × coverage per sample; *n* = 66 lines; accession number PRJNA658680) which was used to assemble and annotate genomes, and call mutations (see Supplemental Methods as well as Ho et al. 2019, 2020, 2021 ; Ho and Schaack 2021 for details on experimental procedures and variant calling).

Expression data were obtained from publicly available RNA-seq datasets in the Short Read Archive housed at NCBI (Bioproject PRJNA 453118 from *D. magna* neonates [48 to 72 h old; Russo et al. 2018], Bioproject PRJEB39239 from 5-d-old juveniles [Poulsen et al. 2021], Bioproject PRJNA326660 [from 21- to 22-d-old adults; Giraudo et al. 2017]; see Supplemental Methods for details of read filtering and mapping). These data made it possible to comprehensively assess the relationship between expression and mutation among genotypes across a latitudinal gradient, among developmental stages (neonates, juveniles, and adults), among environmental conditions (with or without an anticorrosive agent and common chemical contaminant found in freshwater called 1H-benzotriazole; included in Bioproject PRJNA326660; Giraudo et al. 2017), and between mutation types (base substitutions and insertion/deletions). In addition, data from *D. pulex* MA lines (Flynn et al. 2017) and germline-enriched (ovary) RNA-seq libraries (Bioproject PRJDB3265; Toyota et al. 2015) were publicly available, and aligned to the TCO reference genome (Colbourne et al. 2011; see Supplemental Methods).

For each genotype, we mapped mutations (base substitutions, insertions, and deletions; *n* = 656 [a range of 39 to 136 mutations per genotype]) and RNA-seq reads to compare mean expression levels in windows with mutations to all windows using permutation tests to assess significance. To quantify expression depth and breadth genome-wide, Bedtools (Quinlan and Hall 2010) was used to convert the read alignments (BAM file format) into bed files of coverage per base pair (using the “genomecov” command) using a sliding window. We used 1 Kb windows (sliding in 100 bp increments) and 10 Kb windows (sliding in 1 Kb increments) using the “make windows” and “coverageBed” commands to ensure our observations did not depend on window size. For each window, we quantified expression across the genome via two metrics: expression depth (transcripts per million [TPM]) and expression breadth (percent of bases covered by at least one RNA-seq read). Regions of the genome that were masked prior to mutation calling were also masked prior to calculating expression depth. The expression data are non-normally distributed, yet nonparametric tests do not perform well with large sample sizes (Fagerland 2012), so we used permutation tests to test for differences between windows containing mutations and all windows. Permutation tests were performed in R using custom scripts (see Supplemental Appendix). All *P* values were calculated as the fraction of the 10,000 permutations for which the median expression was greater than the median expression observed in mutation-containing windows. To control for the potential influence of GC-content, we repeated the permutation tests with only windows containing 30% to 50% GC, a range that includes >85% of all windows but for which GC-content is indistinguishable between the mutation-containing windows and the full set of windows. Finally, in addition to checking if mutation-overlapping windows exhibit higher expression, we binned all 10 Kb windows per genome into quartiles based on expression levels (expression depth [TPM]) and counted the fraction of mutation-overlapping windows that fell into each quartile.

## Supplementary Material

evag021_Supplementary_Data

## Data Availability

All sequence data have been deposited at NCBI (PRJNA658680).
