# Supplementary material for: Spontaneous Mutations Occur More in Highly Transcribed Regions in Daphnia
Source: Genome Biol Evol. 2026 Jan 27;18(2):evag021. doi: 10.1093/gbe/evag021 (PMC12915781; doi:10.1093/gbe/evag021)
Supplement: evag021_Supplementary_Data [file evag021_supplementary_data.pdf]

SUPPLEMENTAL MATERIALS

Supplemental Methods

Supplemental Figures S1-S10

Supplemental Tables S1, S2, and S3 (also available as workbook)

Supplemental References

Appendix

Supplemental Methods

Three genotypes from each of three populations (Finland, Germany, and Israel) were used to initiate laboratory stocks. From these lab stocks, mutation accumulation (MA) lines (n = 5-12 per genotype). Lineages were initiated and propagated in parallel for 9 genotypes (referred to as FA, FB, FC, GA, GB, GC, IA, IB, and IC). Tissue from each line (MAs and ECs) was frozen regularly during the mutation accumulation period; the average number of generations across MA lines was 12 and the experiment ran for approximately 30 months in total. Starting controls (SCs) were also selected (immediate descendants of the starting clones which were frozen and later sequenced) for each of the 9 genotypes.

The MA lines from each genotype were maintained as single individuals or large populations in 250 mL beakers containing 175-200 mL or 3.5 L jars containing 3 L of Aachener Daphnien Medium (ADaM; Klüttgen et al. 1994), respectively. All lines were maintained under a constant photoperiod (16L:8D) and temperature (18° C), and fed *Scenedesmus obliquus* (3 times per week *ad libitum*). While selection is permitted to act in the large population ECs, the single-progeny descent used to propagate the MA lines maximizes chance and minimizes selection, and thus allows for the accumulation of mutations. The experimental protocols and full details on all variant calling procedures used here have been described previously (Ho et al. 2019, 2020, 2021a). If MA lines went extinct, tissue from the most recent regular tissue collection was used for DNA extraction and sequencing.

At the end of the mutation accumulation period, the 9 SCs, 66 MA lines, and 18 ECs were sequenced. Five asexually-produced clonal individuals from each SC line, all

derived MA lines, and the extant control lines were flash frozen for DNA. Libraries were used to generate approximately 50x coverage genome-wide for each sample. Paired-end reads were used to assemble and annotate genomes, and mutations were called by mapping reads to each individual assembly. Because MA lines (Flynn et al. 2017) and germline-enriched (ovary) RNA-seq libraries (Toyota et al. 2015) have been generated for *Daphnia pulex*, a congener of *D. magna*, we also assessed the association between transcription and mutation using these data (see Supplemental Tabel S2).

Mutation calling and quantification of gene expression

To reduce reference bias, we built reference-guided assemblies for each of the 9 genotypes (using the WGS from each SC). WGS reads from each sample were processed by trimming adaptor sequences (k=23, ktrim=r, mink=4, hdist=1, tpe, tbo), merging overlapping pairs (vstrict=t), and quality filtering (qtrim = rl, trimq=20, minlen=50) with BBTools (Bushnell et al. 2017). First pass reference-guided *de novo* assemblies were performed with SPAdes (using the *trusted-contigs* option; Bankevich et al. 2012). The *D. magna* reference used to guide the assembly was provided by Peter Fields and Dieter Ebert (*personal communication*). To remove haplotigs potentially derived from assembling heterozygous regions into alternate alleles, we collapsed the filtered assemblies with *redundans* by implementing information from paired-end reads, merged reads, and the *D. magna* reference genome for scaffolding (Pryszcz and Gabaldón 2016). We then mapped the processed reads of each SC dataset onto the collapsed assemblies with BWA-MEM v0.7.17 (Li and Durbin 2010).

Contigs were removed if they possessed an average depth of coverage <5 or were shorter than 5 kb.

To compare expression in genic versus non-genic regions, we annotated the genome. Annotation was performed separately for each of the nine assemblies. Gene model prediction was conducted by the MAKER pipeline (Cantarel et al. 2008), integrating *ab-initio* prediction with transcripts and protein datasets. For MAKER, we initially constructed gene models by aligning *Daphnia* transcriptome datasets (wfleabase: *D. galeata, D. magna, D. pulex*) and *Crustacea* proteome datasets (Uniprot database: *Penaeus vannamei, Armadillidium nasatum, Portunus trituberculatus, Tigriopus californicus, Amphibalanus amphitrite, Armadillidium vulgare, D. pulex, D. magna*) to the repeat-masked assembly. Repeats were identified by RepeatModeler (Smit & Hubley 2008) and RepeatMasker (Smit et al. 2013) (described below). We then filtered for gene models with AED values <= 0.25 and used these as input for the *ab-initio* gene predictors AUGUSTUS (Stanke et al. 2008) and SNAP (Korf 2004). The next round of MAKER then incorporated *ab-initio* prediction with the alignment evidence in the first round. We repeated this process for a total of three rounds and identified 15228 to 22072 genes across the nine assemblies. We further processed these gene annotations to identify single-copy genes. If genes overlapped one another, we only kept the gene with the lower AED value. We then performed a BLAST (Altschul et al. 1990) search of each gene set to itself. If any gene had at least 50% of its length matched with another gene and an e-value of 10^{-10}, it was considered a multi-copy gene and discarded. After these filters, we recovered 7720 to 11340 single copy genes across the nine assemblies.

Expression data

RNA-seq data were obtained from the NCBI SRA from projects measuring expression in *D. magna* neonates (48-72 hours old; Bioproject PRJNA 453118; Russo et al. 2018), juveniles (5 days old; Bioproject PRJEB39239; Poulsen et al. 2021), and adults (21-22 days old; Bioproject PRJNA326660; Giraudo et al. 2017) (Supplemental Table 2). For quantification of development-specific expression (neonates, juveniles, and adults), no-treatment control libraries were selected from each project because these best matched the conditions under which the MA lines were reared. The four no 1H-benzotriazole (BTR) control libraries from Giraudo et al. (2017) were used to represent the adult stage. To assess expression in adults under different growth conditions, the BTR treated animals were compared to their corresponding control libraries (Giraudo et al. 2017). In total, we analyzed three control libraries (CDDP controls) for neonates, five control libraries for juveniles, and four control libraries plus four BTR treatment libraries for adults. Additionally, three RNA-seq libraries generated from ovaries of *D. pulex* reared under long day (14hr) conditions were also obtained from the NCBI SRA (Bioproject PRJDB3265; Toyota et al. 2015).

For all samples, fastq files were filtered to remove technical contaminants (adapter and primer sequences) and low quality reads, and trimmed on quality using Trimmomatic (Bolger et al., 2014) with the following settings: ILLUMINACLIP:TruSeq3-PE-2.fa:2:30:10:2:keepBothReads LEADING:3 TRAILING:3 MINLEN:36. Surviving reads for the *D. magna* libraries were then mapped to each of the 9 *D. magna* extant control reference genomes using HISAT2 (Kim et al., 2019) as follows: the reference genome was indexed using “hisat2-build” with default parameters.

RNA-seq reads were then mapped to the indexed reference genome with the following: hisat2 -p 8 --dta -x [index base name] -1 [read 1 fastq file] -2 [read 2 fastq file] -S [name of output file in SAM format]. Surviving reads from the *D. pulex* libraries were mapped to the *D. pulex* reference genome (TCO; dpulex_jgi060905_evenline.fa; (Colbourne et al., 2011)) which was downloaded from wFleaBase (http://wfleabase.org/genome/Daphnia_pulex/current/genome-assembly/) on May 19, 2021, using the same HISAT2 parameters. For all libraries, Bedtools (Quinlan and Hall, 2010) was used to convert the resulting BAM files into bed files of coverage either per base pair (using the 'genomecov' command) or for 10 Kb windows sliding in 1 Kb increments (using the 'make windows' and 'coverageBed' commands) across the relevant genome. In each case, loci masked from the genome assemblies prior to mutation calling were also excluded from the expression analyses.

Expression was quantified by two different metrics - 'expression depth' (Transcripts Per Million [TPM]), and 'expression breadth' (percent of base pairs in a window overlapped by one or more reads). For each developmental stage or treatment, the average value of biological replicates was calculated for each window or base pair. In addition to measuring expression per-base and per-sliding window, expression was also quantified per gene by mapping reads to genic regions as delineated in GFF3 annotation files using HTSeq. For each RNA-Seq library, raw counts per gene were converted to TPM normalized values prior to averaging replicates. Expression-related analyses were performed on Jetstream2 virtual machines provided by ACCESS (Boerner et al. 2023).

Statistical Analyses

Welch's T-tests were used to test for differences in expression in mutated windows vs. all genomic windows after combining genotypes, developmental stages and treatments (Fig. 1). Permutation tests were performed in R using custom scripts (see Appendix A). For each test, a number of windows equal to the number of mutation-containing windows was randomly sampled, without replacement, from the full set of windows for the genome, and the median expression levels (depth and breadth) calculated. This process was repeated 10,000 times. P values were calculated as the fraction of the 10,000 permutations for which median expression was greater than the median expression observed in mutation-containing windows. To assess the influence of GC content on expression, we repeated the permutation tests restricting the analysis to windows with GC contents of 30-50%, a range that includes >85% of all windows but for which GC content is indistinguishable between the mutation-containing windows and the full set of windows.

For each genotype, we mapped mutations (base substitutions, insertions, and deletions; n = 656, ranging from 39-136 per genotype; see Supplemental Table S3) and RNA-seq reads to compare mean expression levels in windows with and without mutations, using permutation tests to assess significance. We quantified expression using 10 Kb sliding windows across the genome via two metrics: expression depth (transcripts per million [TPM]) and expression breadth (percent of bases covered by at least one RNA-seq read). Differences in expression levels between windows with and without mutations were tested across genotypes (nine total from Finland, Germany, and Israel), developmental stages (neonates, juveniles and adults), and environmental conditions (with or without 1H-benzotriazole [B]).

Supplemental Tables

Table S1. Means, medians, standard errors and P-values from permutation tests for all comparisons between windows containing mutations and all windows. For each comparison, p = fraction of median values from 10,000 permutations >= the median value of mutation-overlapping windows. Non-significant tests (p > 0.05) are shown in red.

		Expression breadth (% coverage)							Expression depth (TPM)						
		All Windows				Mutation-containing Windows			All Windows				Mutation-containing Windows		
Genotype	Stage	Median	Mean	Std Dev	P	Median	Mean	Std Dev	Median	Mean	Std Dev	P	Median	Mean	Std Dev
Windows Genome-Wide															
FA	adult	0.68	0.56	0.37	**0.0371**	0.72	0.61	0.34	1.45	9.08	66.3	**0.0177**	2.02	9.61	39.53
FA	adult + BTR	0.7	0.57	0.37	**0.0400**	0.74	0.64	0.34	0.87	9.08	91.62	0.3020	0.97	6.42	20.61
FA	juvenile	0.74	0.6	0.36	**0.0115**	0.79	0.66	0.33	1.59	9.06	46.01	**0.0304**	2.16	9.86	37.01
FA	neonate	0.69	0.58	0.38	**0.0000**	0.82	0.68	0.33	1.59	9.1	49.21	**0.0343**	2.1	12.47	61.35
FB	adult	0.67	0.56	0.37	**0.0000**	0.88	0.74	0.28	1.46	9.06	62.35	**0.0000**	3.53	9	14.69
FB	adult + BTR	0.69	0.57	0.38	**0.0000**	0.88	0.76	0.27	0.78	9.01	104.8	**0.0000**	2.26	9.11	18.87
FB	juvenile	0.73	0.6	0.36	**0.0000**	0.84	0.75	0.25	1.43	8.99	56.52	**0.0000**	3.84	9.67	16.57
FB	neonate	0.69	0.58	0.38	**0.0000**	0.86	0.75	0.29	1.55	8.88	49.93	**0.0000**	4.03	9.13	15.13
FC	adult	0.67	0.56	0.38	**0.0000**	0.77	0.66	0.34	1.51	9.03	55.65	**0.0000**	3.2	11.49	32.36
FC	adult + BTR	0.69	0.57	0.37	**0.0000**	0.77	0.66	0.33	0.89	9.03	92.3	**0.0000**	2.19	11.45	37
FC	juvenile	0.74	0.6	0.36	**0.0000**	0.85	0.71	0.33	1.54	8.98	41.97	**0.0000**	3.42	11.29	25.83
FC	neonate	0.68	0.57	0.38	**0.0000**	0.8	0.67	0.33	1.64	9.07	44.24	**0.0000**	3.43	11.51	31.75
GA	adult	0.67	0.56	0.37	**0.0000**	0.75	0.64	0.34	1.64	9.81	77.66	**0.0000**	2.95	6.7	15
GA	adult + BTR	0.69	0.57	0.37	**0.0000**	0.77	0.65	0.34	0.93	9.86	111.64	**0.0000**	2.32	7.12	25.69
GA	juvenile	0.74	0.6	0.36	**0.0000**	0.81	0.67	0.33	1.71	9.86	61.29	**0.0000**	3.37	8.18	25.46
GA	neonate	0.77	0.61	0.38	**0.0226**	0.8	0.68	0.33	1.41	9.98	98.12	**0.0000**	2.79	6.99	18.98
GB	adult	0.68	0.56	0.38	0.0516	0.73	0.62	0.34	1.68	9.55	54.46	0.1412	2.04	12.95	52.31
GB	adult + BTR	0.7	0.58	0.38	0.0066	0.76	0.64	0.34	0.93	9.62	112.62	**0.0151**	1.45	10.13	26.78
GB	juvenile	0.74	0.6	0.36	0.0008	0.8	0.66	0.34	1.72	9.57	51.67	**0.0214**	2.4	13.31	43.54
GB	neonate	0.78	0.62	0.38	0.0668	0.81	0.68	0.34	1.61	9.87	80.91	0.3541	1.73	11.35	41.42
GC	adult	0.66	0.55	0.38	**0.0000**	0.78	0.67	0.32	1.62	10.11	69.05	**0.0000**	4.66	13.34	29.62
GC	adult + BTR	0.68	0.56	0.38	**0.0000**	0.79	0.68	0.31	0.82	10.06	142.72	**0.0000**	2.17	11.43	29.1
GC	juvenile	0.73	0.59	0.37	**0.0000**	0.83	0.69	0.32	1.66	10.06	59.2	**0.0000**	4.55	13.55	30.5
GC	neonate	0.74	0.6	0.38	**0.0000**	0.86	0.7	0.33	1.56	9.95	72.47	**0.0000**	4.19	12.28	27.29
IA	adult	0.67	0.56	0.38	0.0881	0.71	0.63	0.32	1.4	9.46	138.37	**0.0001**	2.65	9.53	16.23
IA	adult + BTR	0.69	0.57	0.37	0.3982	0.7	0.63	0.32	0.72	9.54	178.31	**0.0000**	1.83	8.01	15.69
IA	juvenile	0.73	0.59	0.36	**0.0000**	0.81	0.7	0.31	1.48	9.66	141.96	**0.0002**	2.91	10.33	19.99
IA	neonate	0.77	0.61	0.38	**0.0000**	0.85	0.71	0.32	1.36	9.24	130.42	**0.0005**	2.45	9.92	17.82
IB	adult	0.64	0.54	0.38	**0.0000**	0.77	0.65	0.35	1.55	9.8	53.15	**0.0315**	2.36	10.42	24.02
IB	adult + BTR	0.66	0.55	0.38	**0.0000**	0.79	0.63	0.37	0.94	9.81	84.27	**0.0090**	1.73	6.17	10.1
IB	juvenile	0.71	0.58	0.37	**0.0343**	0.76	0.66	0.34	1.73	9.81	60.45	0.1380	2.21	8.35	18.71
IB	neonate	0.75	0.6	0.38	**0.0000**	0.9	0.74	0.31	1.6	9.81	58.89	**0.0033**	2.27	7.86	17.54
IC	adult	0.66	0.55	0.38	**0.0000**	0.78	0.68	0.31	1.61	9.55	44.26	**0.0000**	5.42	15.92	43.27
IC	adult + BTR	0.69	0.57	0.37	**0.0000**	0.8	0.71	0.28	0.83	9.55	115.9	**0.0000**	3.26	9.34	17.16
IC	juvenile	0.73	0.59	0.36	**0.0000**	0.82	0.7	0.28	1.6	9.59	62.74	**0.0000**	4.75	14.31	28.68
IC	neonate	0.77	0.61	0.38	**0.0000**	0.89	0.77	0.28	1.54	9.4	59.31	**0.0000**	4.83	12.4	23.73
Windows with 30-50% GC Content															
FA	adult	0.72	0.6	0.36	0.4454	0.72	0.62	0.34	1.9	9.4	62.72	0.4483	1.93	9.83	40.22
FA	adult + BTR	0.74	0.6	0.36	0.4528	0.74	0.64	0.33	1.19	9.22	81.51	0.8696	0.97	6.59	20.95
FA	juvenile	0.78	0.63	0.35	0.2893	0.79	0.67	0.32	2.09	9.45	43.19	0.5746	2.03	10.07	37.66
FA	neonate	0.76	0.63	0.36	**0.0001**	0.82	0.69	0.33	2.05	9.54	49.33	0.5709	2	12.75	62.43
FB	adult	0.71	0.59	0.36	**0.0000**	0.88	0.74	0.29	1.94	9.63	62.23	**0.0005**	3.43	8.63	13.97
FB	adult + BTR	0.73	0.6	0.36	**0.0000**	0.88	0.76	0.27	1.11	9.27	96.51	**0.0003**	2.21	9	19.02
FB	juvenile	0.77	0.63	0.35	**0.0000**	0.84	0.75	0.25	1.9	9.5	55.02	**0.0000**	3.74	9.35	16.19
FB	neonate	0.76	0.63	0.36	**0.0000**	0.88	0.77	0.28	2.05	9.47	51.06	**0.0002**	3.96	8.67	14.24
FC	adult	0.72	0.6	0.36	**0.0001**	0.78	0.67	0.33	2.09	9.32	43.92	**0.0005**	3.43	12.07	33.32
FC	adult + BTR	0.74	0.61	0.36	**0.0000**	0.77	0.67	0.33	1.28	9.04	65.42	**0.0000**	2.36	12.05	38.12
FC	juvenile	0.78	0.64	0.35	**0.0000**	0.86	0.72	0.32	2.12	9.48	40.56	**0.0000**	3.57	11.87	26.56
FC	neonate	0.76	0.63	0.36	**0.0005**	0.81	0.69	0.32	2.25	9.54	40.69	**0.0000**	3.61	12.09	32.7
GA	adult	0.72	0.6	0.36	0.0583	0.75	0.65	0.32	2.15	10.06	70.94	**0.0005**	3.15	6.94	15.34
GA	adult + BTR	0.74	0.61	0.36	**0.0099**	0.77	0.66	0.33	1.28	9.58	84.93	**0.0000**	2.39	7.39	26.37
GA	juvenile	0.78	0.64	0.35	**0.0012**	0.82	0.68	0.32	2.24	10.3	56.36	**0.0000**	3.58	8.49	26.12
GA	neonate	0.81	0.65	0.36	0.5437	0.81	0.7	0.32	1.86	10.03	73.45	**0.0000**	2.97	7.26	19.45
GB	adult	0.74	0.61	0.36	0.6675	0.73	0.63	0.33	2.3	9.83	47.04	0.6873	2.13	13.4	53.72
GB	adult + BTR	0.75	0.62	0.36	0.2859	0.76	0.64	0.34	1.34	9.24	75	0.2111	1.55	10.37	27.46
GB	juvenile	0.78	0.64	0.35	0.1022	0.8	0.67	0.33	2.32	9.92	46.94	0.3608	2.45	13.77	44.69
GB	neonate	0.82	0.66	0.36	0.5955	0.82	0.69	0.33	2.18	9.91	62.16	0.9380	1.73	11.78	42.53
GC	adult	0.73	0.6	0.37	**0.0002**	0.79	0.7	0.3	2.37	10.38	54.15	**0.0005**	4.8	14.09	30.42
GC	adult + BTR	0.74	0.61	0.36	**0.0003**	0.81	0.71	0.29	1.29	9.61	102.39	**0.0001**	2.38	12.13	29.9
GC	juvenile	0.78	0.63	0.35	**0.0000**	0.84	0.71	0.3	2.39	10.53	53.41	**0.0000**	4.88	14.34	31.31
GC	neonate	0.8	0.65	0.36	**0.0000**	0.87	0.71	0.32	2.28	10.2	60.5	**0.0000**	4.31	12.97	28.03
IA	adult	0.71	0.59	0.37	0.3496	0.72	0.64	0.32	1.8	9.84	144.58	**0.0052**	2.67	9.61	16.3
IA	adult + BTR	0.73	0.6	0.36	0.8266	0.7	0.64	0.32	0.98	9.6	179.88	**0.0001**	1.84	8.07	15.76
IA	juvenile	0.76	0.62	0.35	**0.0031**	0.81	0.7	0.31	1.9	10.08	149	**0.0013**	2.93	10.42	20.09
IA	neonate	0.8	0.64	0.37	**0.0037**	0.85	0.72	0.32	1.76	9.83	138.27	**0.0162**	2.49	10.01	17.9
IB	adult	0.7	0.58	0.37	**0.0000**	0.82	0.67	0.34	2.14	10.26	43.76	0.5915	2.03	9.72	22.52
IB	adult + BTR	0.72	0.59	0.36	**0.0000**	0.82	0.64	0.37	1.38	10.03	75.01	0.3143	1.55	5.5	8.75
IB	juvenile	0.76	0.62	0.35	0.2396	0.77	0.68	0.34	2.35	10.16	51.08	0.7060	2.1	7.7	16.87
IB	neonate	0.8	0.64	0.37	**0.0000**	0.9	0.76	0.29	2.22	10.13	47.59	0.5148	2.2	7.45	15.98
IC	adult	0.71	0.59	0.36	**0.0008**	0.78	0.68	0.3	2.06	9.83	40.92	**0.0005**	5.4	16.09	43.82
IC	adult + BTR	0.73	0.6	0.36	**0.0002**	0.8	0.72	0.28	1.12	8.95	74.68	**0.0005**	3.31	9.53	17.35
IC	juvenile	0.77	0.62	0.35	**0.0039**	0.82	0.7	0.28	2.04	9.73	52.33	**0.0000**	4.74	14.46	29.02
IC	neonate	0.8	0.64	0.37	**0.0000**	0.89	0.77	0.28	2	9.49	45.48	**0.0000**	4.87	12.52	23.99

Table S2. Details about the RNA-Seq datasets for *D. magna* and *D. pulex* obtained from the Sequence Read Archive (SRA) and used to quantify gene expression.

BioProject	Run	Species	Tissue[1]	Stage	Age	Sample	Reads
PRJNA453	SRR7058642	*D. magna*	Whole body	Neonate	48-72 hr	untreated control, CDDP	PE 2x100
	SRR7058645	*D. magna*	Whole body	Neonate	48-72 hr	untreated control, CDDP	PE 2x100
	SRR7058646	*D. magna*	Whole body	Neonate	48-72 hr	untreated control, CDDP	PE 2x100
PRJEB392	ERR4318984	*D. magna*	Whole body	Juvenile	5 d	Untreated control	SE 1x50
	ERR4318983	*D. magna*	Whole body	Juvenile	5 d	Untreated control	SE 1x50
	ERR4318982	*D. magna*	Whole body	Juvenile	5 d	Untreated control	SE 1x50
	ERR4318981	*D. magna*	Whole body	Juvenile	5 d	Untreated control	SE 1x50
	ERR4318980	*D. magna*	Whole body	Juvenile	5 d	Untreated control	SE 1x50
PRJNA326	SRR3996388	*D. magna*	Whole body	Adult	21-22 d	BTR_CTRL	PE 2x100
	SRR3996389	*D. magna*	Whole body	Adult	21-22 d	BTR_CTRL	PE 2x100
	SRR3996401	*D. magna*	Whole body	Adult	21-22 d	BTR_CTRL	PE 2x100
	SRR3996409	*D. magna*	Whole body	Adult	21-22 d	BTR_CTRL	PE 2x100
	SRR3996410	*D. magna*	Whole body	Adult	21-22 d	BTR_2000	PE 2x100
	SRR3996411	*D. magna*	Whole body	Adult	21-22 d	BTR_2000	PE 2x100
	SRR3996412	*D. magna*	Whole body	Adult	21-22 d	BTR_2000	PE 2x100
	SRR3996414	*D. magna*	Whole body	Adult	21-22 d	BTR_2000	PE 2x100
PRJDB326	DRR024779	*D. pulex*	Ovary	Adult		Long day	PE 2x100
	DRR024785	*D. pulex*	Ovary	Adult		Long day	PE 2x100
	DRR024791	*D. pulex*	Ovary	Adult		Long day	PE 2x100

Table S3. Summary of mutations that occurred in *D. magna* mutation accumulation lines (reported previously) that were used to establish mutation-contaning windows in this analysis.

Genotype	Deletions	Insertions	Base substitutions	Total
FA	5	10	67	82
FB	18	3	20	41
FC	14	14	108	136
GA	13	5	102	120
GB	7	6	50	63
GC	4	5	54	63
IA	6	3	47	56
IB	5	3	31	39
IC	4	2	49	55
**Grand Total**	**76**	**51**	**528**	**655**

Genotype	Scaffold	Mutation	Start	End
FA	scaffold00001	Base sub	1147935	1147935
FA	scaffold00001	Base sub	3743064	3743064
FA	scaffold00001	Base sub	3781249	3781249
FA	scaffold00001	Base sub	6553548	6553548
FA	scaffold00001	Base sub	995067	995067
FA	scaffold00001	Insertion	4220164	4220164
FA	scaffold00002	Base sub	1480739	1480739
FA	scaffold00002	Base sub	2918266	2918266
FA	scaffold00002	Base sub	3052943	3052943
FA	scaffold00002	Base sub	4953268	4953268
FA	scaffold00002	Base sub	714173	714173
FA	scaffold00003	Base sub	1192630	1192630

FA	scaffold00003	Base sub	43069	43069
FA	scaffold00003	Base sub	968015	968015
FA	scaffold00003	Insertion	366021	366021
FA	scaffold00004	Base sub	144341	144341
FA	scaffold00004	Base sub	2339048	2339048
FA	scaffold00004	Base sub	3058551	3058551
FA	scaffold00004	Base sub	3463343	3463343
FA	scaffold00005	Base sub	2043123	2043123
FA	scaffold00005	Base sub	2970669	2970669
FA	scaffold00006	Base sub	1668514	1668514
FA	scaffold00006	Base sub	331939	331939
FA	scaffold00006	Base sub	397877	397877
FA	scaffold00007	Base sub	656455	656455
FA	scaffold00009	Base sub	1775199	1775199
FA	scaffold00009	Base sub	662226	662226
FA	scaffold00009	Deletion	1039312	1039330
FA	scaffold00010	Base sub	1242346	1242346
FA	scaffold00010	Base sub	821340	821340
FA	scaffold00011	Base sub	224938	224938
FA	scaffold00011	Insertion	2597918	2597918
FA	scaffold00012	Base sub	484109	484109
FA	scaffold00012	Base sub	729683	729683
FA	scaffold00012	Base sub	797205	797205
FA	scaffold00013	Base sub	1206773	1206773
FA	scaffold00016	Base sub	163868	163868
FA	scaffold00016	Base sub	634839	634839
FA	scaffold00017	Base sub	1685926	1685926
FA	scaffold00017	Base sub	1766937	1766937
FA	scaffold00017	Base sub	1939040	1939040
FA	scaffold00017	Deletion	1296118	1296119
FA	scaffold00019	Deletion	1648313	1648316
FA	scaffold00020	Base sub	308526	308526
FA	scaffold00021	Base sub	1115664	1115664
FA	scaffold00022	Base sub	946347	946347
FA	scaffold00022	Deletion	954922	954923
FA	scaffold00023	Base sub	1785525	1785525
FA	scaffold00024	Base sub	493502	493502
FA	scaffold00025	Base sub	1212947	1212947
FA	scaffold00027	Base sub	1325	1325
FA	scaffold00027	Base sub	352870	352870
FA	scaffold00028	Base sub	532552	532552

FA	scaffold00029	Insertion	696389	696389
FA	scaffold00030	Base sub	1307594	1307594
FA	scaffold00031	Base sub	662163	662163
FA	scaffold00031	Insertion	810825	810825
FA	scaffold00034	Base sub	991578	991578
FA	scaffold00035	Base sub	991268	991268
FA	scaffold00036	Base sub	8944	8944
FA	scaffold00038	Insertion	638316	638316
FA	scaffold00039	Base sub	555986	555986
FA	scaffold00039	Insertion	264709	264709
FA	scaffold00040	Base sub	590028	590028
FA	scaffold00050	Base sub	162358	162358
FA	scaffold00058	Base sub	270676	270676
FA	scaffold00064	Base sub	324471	324471
FA	scaffold00072	Base sub	303571	303571
FA	scaffold00076	Base sub	9491	9491
FA	scaffold00081	Insertion	148607	148607
FA	scaffold00103	Base sub	51584	51584
FA	scaffold00498	Base sub	5390	5390
FA	scaffold01050	Base sub	32282	32282
FA	scaffold01256	Base sub	18069	18069
FA	scaffold01323	Insertion	9982	9982
FA	scaffold01381	Deletion	104094	104099
FA	scaffold01628	Base sub	2485	2485
FA	scaffold02208	Base sub	14724	14724
FA	scaffold02350	Insertion	25435	25435
FA	scaffold02742	Base sub	24458	24458
FA	scaffold03023	Base sub	20723	20723
FA	scaffold03962	Base sub	22931	22931
FB	scaffold00001	Base sub	1532285	1532285
FB	scaffold00001	Deletion	4700422	4700426
FB	scaffold00002	Deletion	2343555	2343559
FB	scaffold00003	Base sub	535479	535479
FB	scaffold00003	Deletion	1816153	1816162
FB	scaffold00005	Deletion	1654098	1654101
FB	scaffold00005	Deletion	3099076	3099079
FB	scaffold00009	Deletion	2366863	2366869
FB	scaffold00011	Base sub	1910042	1910042
FB	scaffold00011	Base sub	361969	361969
FB	scaffold00011	Base sub	684606	684606
FB	scaffold00011	Base sub	684607	684607

FB	scaffold00012	Base sub	790841	790841
FB	scaffold00013	Base sub	1197173	1197173
FB	scaffold00013	Deletion	1710063	1710066
FB	scaffold00015	Base sub	1478794	1478794
FB	scaffold00016	Base sub	8166	8166
FB	scaffold00017	Deletion	2136731	2136786
FB	scaffold00018	Deletion	1633575	1633578
FB	scaffold00020	Deletion	1570369	1570371
FB	scaffold00023	Base sub	121347	121347
FB	scaffold00023	Base sub	40470	40470
FB	scaffold00024	Base sub	1026777	1026777
FB	scaffold00024	Base sub	695295	695295
FB	scaffold00030	Deletion	739387	739391
FB	scaffold00030	Deletion	739392	739394
FB	scaffold00031	Base sub	13866	13866
FB	scaffold00035	Insertion	199960	199960
FB	scaffold00040	Base sub	50936	50936
FB	scaffold00040	Insertion	436423	436423
FB	scaffold00043	Deletion	297687	297695
FB	scaffold00062	Deletion	157960	157962
FB	scaffold00071	Insertion	366727	366727
FB	scaffold00748	Deletion	2017	2032
FB	scaffold01360	Deletion	15279	15283
FB	scaffold02179	Base sub	10509	10509
FB	scaffold02749	Base sub	11015	11015
FB	scaffold03103	Base sub	20470	20470
FB	scaffold03247	Deletion	31256	31258
FB	scaffold03751	Base sub	4790	4790
FB	scaffold04227	Deletion	135080	135082
FC	scaffold00001	Base sub	1503046	1503046
FC	scaffold00001	Base sub	1742254	1742254
FC	scaffold00001	Base sub	2124843	2124843
FC	scaffold00001	Base sub	2157413	2157413
FC	scaffold00001	Base sub	2745592	2745592
FC	scaffold00001	Base sub	2973645	2973645
FC	scaffold00001	Base sub	3692100	3692100
FC	scaffold00001	Base sub	4150875	4150875
FC	scaffold00001	Base sub	4226848	4226848
FC	scaffold00001	Base sub	4337975	4337975
FC	scaffold00001	Base sub	468213	468213
FC	scaffold00001	Base sub	5060948	5060948

FC	scaffold00001	Base sub	639158	639158
FC	scaffold00001	Base sub	751843	751843
FC	scaffold00001	Base sub	948037	948037
FC	scaffold00002	Base sub	1020310	1020310
FC	scaffold00002	Base sub	1285905	1285905
FC	scaffold00002	Base sub	3136674	3136674
FC	scaffold00002	Base sub	3780828	3780828
FC	scaffold00002	Base sub	4367410	4367410
FC	scaffold00002	Base sub	5196124	5196124
FC	scaffold00002	Base sub	850208	850208
FC	scaffold00002	Deletion	2546615	2546624
FC	scaffold00003	Base sub	1047769	1047769
FC	scaffold00003	Base sub	2213003	2213003
FC	scaffold00004	Base sub	2096945	2096945
FC	scaffold00004	Base sub	3147286	3147286
FC	scaffold00004	Deletion	2039970	2039973
FC	scaffold00005	Base sub	1356571	1356571
FC	scaffold00005	Base sub	1504425	1504425
FC	scaffold00005	Base sub	1528673	1528673
FC	scaffold00005	Base sub	1836809	1836809
FC	scaffold00005	Deletion	1655082	1655087
FC	scaffold00005	Deletion	4007730	4007731
FC	scaffold00005	Insertion	1858402	1858402
FC	scaffold00005	Insertion	2352749	2352749
FC	scaffold00006	Base sub	2136823	2136823
FC	scaffold00006	Base sub	269844	269844
FC	scaffold00006	Base sub	3154083	3154083
FC	scaffold00006	Base sub	3323084	3323084
FC	scaffold00006	Base sub	3501284	3501284
FC	scaffold00006	Insertion	3157378	3157378
FC	scaffold00007	Base sub	2223382	2223382
FC	scaffold00008	Base sub	1185931	1185931
FC	scaffold00009	Base sub	1359035	1359035
FC	scaffold00009	Base sub	1860694	1860694
FC	scaffold00009	Base sub	2539146	2539146
FC	scaffold00009	Base sub	394767	394767
FC	scaffold00009	Base sub	791383	791383
FC	scaffold00010	Base sub	1256540	1256540
FC	scaffold00010	Base sub	2409155	2409155
FC	scaffold00011	Base sub	1081734	1081734
FC	scaffold00011	Base sub	2028173	2028173

FC	scaffold00012	Base sub	874726	874726
FC	scaffold00013	Base sub	1043837	1043837
FC	scaffold00013	Base sub	1384609	1384609
FC	scaffold00014	Base sub	1975861	1975861
FC	scaffold00014	Base sub	291262	291262
FC	scaffold00014	Base sub	291267	291267
FC	scaffold00014	Insertion	939108	939108
FC	scaffold00015	Base sub	2386874	2386874
FC	scaffold00015	Base sub	638491	638491
FC	scaffold00015	Base sub	643350	643350
FC	scaffold00015	Deletion	799062	799064
FC	scaffold00016	Base sub	1219054	1219054
FC	scaffold00016	Base sub	800284	800284
FC	scaffold00016	Deletion	646521	646528
FC	scaffold00017	Base sub	1651856	1651856
FC	scaffold00017	Base sub	249788	249788
FC	scaffold00017	Base sub	331727	331727
FC	scaffold00017	Base sub	417848	417848
FC	scaffold00017	Base sub	609948	609948
FC	scaffold00017	Deletion	1979830	1979833
FC	scaffold00018	Deletion	1562171	1562174
FC	scaffold00020	Base sub	1435069	1435069
FC	scaffold00021	Deletion	767234	767235
FC	scaffold00022	Base sub	953373	953373
FC	scaffold00023	Base sub	538361	538361
FC	scaffold00024	Base sub	875683	875683
FC	scaffold00024	Insertion	418345	418345
FC	scaffold00029	Base sub	662476	662476
FC	scaffold00030	Base sub	1070101	1070101
FC	scaffold00030	Base sub	305700	305700
FC	scaffold00030	Base sub	833209	833209
FC	scaffold00030	Base sub	941641	941641
FC	scaffold00032	Base sub	452731	452731
FC	scaffold00032	Insertion	414819	414819
FC	scaffold00032	Insertion	815224	815224
FC	scaffold00033	Base sub	325317	325317
FC	scaffold00034	Base sub	968095	968095
FC	scaffold00037	Base sub	70617	70617
FC	scaffold00038	Base sub	317103	317103
FC	scaffold00038	Base sub	320647	320647
FC	scaffold00039	Base sub	52016	52016

FC	scaffold00039	Base sub	651756	651756
FC	scaffold00041	Base sub	60849	60849
FC	scaffold00042	Base sub	469911	469911
FC	scaffold00045	Base sub	352155	352155
FC	scaffold00047	Base sub	106724	106724
FC	scaffold00047	Base sub	293718	293718
FC	scaffold00049	Base sub	195828	195828
FC	scaffold00050	Base sub	105171	105171
FC	scaffold00050	Base sub	500234	500234
FC	scaffold00050	Deletion	405360	405361
FC	scaffold00050	Insertion	107483	107483
FC	scaffold00055	Insertion	57539	57539
FC	scaffold00058	Base sub	335691	335691
FC	scaffold00064	Deletion	113257	113259
FC	scaffold00064	Insertion	114078	114078
FC	scaffold00076	Base sub	48486	48486
FC	scaffold00076	Base sub	78753	78753
FC	scaffold00080	Base sub	38187	38187
FC	scaffold00084	Base sub	146111	146111
FC	scaffold00085	Deletion	163490	163496
FC	scaffold00094	Base sub	75509	75509
FC	scaffold00466	Insertion	2771	2771
FC	scaffold00721	Base sub	10244	10244
FC	scaffold00721	Deletion	10245	10246
FC	scaffold01123	Insertion	3973	3973
FC	scaffold01131	Base sub	35573	35573
FC	scaffold01184	Base sub	42065	42065
FC	scaffold01321	Base sub	19614	19614
FC	scaffold01741	Base sub	2691	2691
FC	scaffold01940	Base sub	112497	112497
FC	scaffold02140	Base sub	10491	10491
FC	scaffold02441	Insertion	2767	2767
FC	scaffold02532	Base sub	27060	27060
FC	scaffold02893	Deletion	4838	4844
FC	scaffold02997	Base sub	32568	32568
FC	scaffold03568	Base sub	6233	6233
FC	scaffold03568	Insertion	6237	6237
FC	scaffold04008	Base sub	6878	6878
FC	scaffold04069	Base sub	8983	8983
FC	scaffold04087	Base sub	36363	36363
FC	scaffold04505	Base sub	8809	8809

FC	scaffold04691	Base sub	5887	5887
GA	scaffold00001	Base sub	2360253	2360253
GA	scaffold00001	Base sub	2531436	2531436
GA	scaffold00001	Base sub	2640965	2640965
GA	scaffold00001	Base sub	3014088	3014088
GA	scaffold00001	Base sub	3566529	3566529
GA	scaffold00001	Base sub	391295	391295
GA	scaffold00001	Base sub	4855623	4855623
GA	scaffold00001	Base sub	6977100	6977100
GA	scaffold00001	Insertion	3287898	3287898
GA	scaffold00002	Base sub	163885	163885
GA	scaffold00002	Base sub	4185070	4185070
GA	scaffold00002	Base sub	970644	970644
GA	scaffold00002	Deletion	2218664	2218666
GA	scaffold00003	Base sub	1136	1136
GA	scaffold00003	Base sub	459170	459170
GA	scaffold00003	Deletion	50469	50476
GA	scaffold00004	Base sub	865163	865163
GA	scaffold00005	Base sub	1435136	1435136
GA	scaffold00005	Base sub	1676346	1676346
GA	scaffold00005	Base sub	2621269	2621269
GA	scaffold00005	Base sub	2893196	2893196
GA	scaffold00005	Base sub	3326034	3326034
GA	scaffold00005	Base sub	3331763	3331763
GA	scaffold00005	Base sub	3541079	3541079
GA	scaffold00005	Base sub	593771	593771
GA	scaffold00005	Base sub	740854	740854
GA	scaffold00005	Deletion	2852294	2852326
GA	scaffold00005	Insertion	3486893	3486893
GA	scaffold00006	Base sub	1671545	1671545
GA	scaffold00006	Base sub	703683	703683
GA	scaffold00006	Base sub	706859	706859
GA	scaffold00007	Base sub	1031987	1031987
GA	scaffold00008	Base sub	463648	463648
GA	scaffold00009	Base sub	1295391	1295391
GA	scaffold00009	Base sub	152207	152207
GA	scaffold00009	Base sub	259098	259098
GA	scaffold00009	Base sub	2800113	2800113
GA	scaffold00009	Base sub	388777	388777
GA	scaffold00010	Base sub	1792712	1792712
GA	scaffold00011	Base sub	2020780	2020780

GA	scaffold00011	Base sub	2327377	2327377
GA	scaffold00011	Deletion	97372	97374
GA	scaffold00012	Base sub	110433	110433
GA	scaffold00012	Base sub	225288	225288
GA	scaffold00012	Base sub	47255	47255
GA	scaffold00013	Deletion	1299996	1299998
GA	scaffold00014	Base sub	1715698	1715698
GA	scaffold00014	Base sub	893789	893789
GA	scaffold00015	Base sub	1118963	1118963
GA	scaffold00015	Base sub	39584	39584
GA	scaffold00015	Base sub	772165	772165
GA	scaffold00015	Deletion	1030967	1030970
GA	scaffold00015	Deletion	8580	8584
GA	scaffold00016	Base sub	1121848	1121848
GA	scaffold00017	Deletion	51062	51071
GA	scaffold00019	Base sub	359526	359526
GA	scaffold00020	Base sub	495271	495271
GA	scaffold00021	Base sub	163720	163720
GA	scaffold00021	Base sub	715437	715437
GA	scaffold00022	Base sub	1279721	1279721
GA	scaffold00022	Base sub	1392742	1392742
GA	scaffold00022	Base sub	48465	48465
GA	scaffold00022	Base sub	943095	943095
GA	scaffold00023	Insertion	474247	474247
GA	scaffold00024	Base sub	594092	594092
GA	scaffold00024	Base sub	602234	602234
GA	scaffold00025	Base sub	643915	643915
GA	scaffold00025	Base sub	803816	803816
GA	scaffold00031	Base sub	918276	918276
GA	scaffold00033	Base sub	247914	247914
GA	scaffold00035	Base sub	669672	669672
GA	scaffold00039	Base sub	225013	225013
GA	scaffold00040	Base sub	198674	198674
GA	scaffold00041	Base sub	206477	206477
GA	scaffold00043	Base sub	112488	112488
GA	scaffold00044	Deletion	610808	610819
GA	scaffold00048	Base sub	323906	323906
GA	scaffold00048	Base sub	323907	323907
GA	scaffold00055	Base sub	43337	43337
GA	scaffold00055	Base sub	43339	43339
GA	scaffold00057	Base sub	422745	422745

GA	scaffold00061	Base sub	299168	299168
GA	scaffold00070	Base sub	171050	171050
GA	scaffold00074	Base sub	241007	241007
GA	scaffold00074	Base sub	57794	57794
GA	scaffold00078	Base sub	79936	79936
GA	scaffold00099	Base sub	87868	87868
GA	scaffold00109	Base sub	18240	18240
GA	scaffold00191	Base sub	4912	4912
GA	scaffold00307	Base sub	2574	2574
GA	scaffold00395	Base sub	19183	19183
GA	scaffold00575	Insertion	8549	8549
GA	scaffold00590	Base sub	13033	13033
GA	scaffold01779	Base sub	3460	3460
GA	scaffold02427	Base sub	3182	3182
GA	scaffold02943	Insertion	4952	4952
GA	scaffold02957	Base sub	14729	14729
GA	scaffold03087	Base sub	10941	10941
GA	scaffold03626	Base sub	3223	3223
GA	scaffold04005	Base sub	71057	71057
GA	scaffold04519	Base sub	125554	125554
GA	scaffold04536	Base sub	400	400
GA	scaffold05032	Base sub	33547	33547
GA	scaffold05478	Deletion	2913	2914
GA	scaffold05515	Base sub	16015	16015
GA	scaffold06006	Base sub	43013	43013
GA	scaffold06009	Base sub	41194	41194
GA	scaffold06035	Deletion	5561	5562
GA	scaffold06045	Base sub	13792	13792
GA	scaffold06078	Base sub	17427	17427
GA	scaffold06314	Base sub	17403	17403
GA	scaffold06398	Base sub	49601	49601
GA	scaffold06718	Deletion	9885	9892
GA	scaffold06880	Base sub	8084	8084
GA	scaffold06907	Base sub	1019	1019
GA	scaffold06933	Deletion	68165	68167
GA	scaffold07541	Base sub	5727	5727
GA	scaffold07683	Base sub	7415	7415
GA	scaffold09544	Base sub	1877	1877
GA	scaffold09544	Base sub	9778	9778
GB	scaffold00001	Base sub	2480054	2480054
GB	scaffold00001	Deletion	5135050	5135055

GB	scaffold00001	Insertion	4702326	4702326
GB	scaffold00002	Base sub	2277845	2277845
GB	scaffold00002	Base sub	2403899	2403899
GB	scaffold00002	Base sub	3890999	3890999
GB	scaffold00003	Base sub	2644393	2644393
GB	scaffold00003	Deletion	694369	694372
GB	scaffold00004	Base sub	2698324	2698324
GB	scaffold00004	Base sub	3228066	3228066
GB	scaffold00005	Base sub	3798696	3798696
GB	scaffold00005	Base sub	3858820	3858820
GB	scaffold00006	Base sub	1075161	1075161
GB	scaffold00007	Base sub	363860	363860
GB	scaffold00007	Insertion	801973	801973
GB	scaffold00009	Base sub	2446810	2446810
GB	scaffold00010	Base sub	1078031	1078031
GB	scaffold00010	Base sub	613200	613200
GB	scaffold00010	Deletion	2356092	2356097
GB	scaffold00010	Insertion	2489959	2489959
GB	scaffold00011	Base sub	1128312	1128312
GB	scaffold00011	Base sub	1264255	1264255
GB	scaffold00011	Deletion	1681104	1681108
GB	scaffold00011	Deletion	2511871	2511877
GB	scaffold00012	Base sub	1692994	1692994
GB	scaffold00012	Base sub	579657	579657
GB	scaffold00013	Base sub	2584965	2584965
GB	scaffold00014	Base sub	1501755	1501755
GB	scaffold00015	Base sub	471382	471382
GB	scaffold00016	Base sub	1666042	1666042
GB	scaffold00017	Base sub	834550	834550
GB	scaffold00017	Base sub	925804	925804
GB	scaffold00017	Insertion	925802	925802
GB	scaffold00018	Base sub	1807973	1807973
GB	scaffold00018	Base sub	2001691	2001691
GB	scaffold00024	Base sub	283047	283047
GB	scaffold00024	Base sub	520032	520032
GB	scaffold00027	Base sub	508443	508443
GB	scaffold00028	Base sub	588522	588522
GB	scaffold00030	Base sub	370640	370640
GB	scaffold00030	Deletion	1117662	1117666
GB	scaffold00031	Base sub	857399	857399
GB	scaffold00032	Base sub	369700	369700

GB	scaffold00033	Base sub	94425	94425
GB	scaffold00033	Deletion	94422	94424
GB	scaffold00036	Base sub	471105	471105
GB	scaffold00036	Base sub	735270	735270
GB	scaffold00038	Base sub	170317	170317
GB	scaffold00038	Base sub	472973	472973
GB	scaffold00044	Base sub	295066	295066
GB	scaffold00046	Base sub	77010	77010
GB	scaffold00056	Base sub	342524	342524
GB	scaffold00056	Insertion	25527	25527
GB	scaffold00198	Insertion	8063	8063
GB	scaffold00704	Base sub	34836	34836
GB	scaffold01312	Base sub	53946	53946
GB	scaffold02503	Base sub	2082	2082
GB	scaffold02556	Base sub	14316	14316
GB	scaffold03481	Base sub	3089	3089
GB	scaffold05362	Base sub	4590	4590
GB	scaffold05377	Base sub	3361	3361
GB	scaffold05424	Base sub	4317	4317
GB	scaffold05795	Base sub	6063	6063
GC	scaffold00001	Base sub	1010706	1010706
GC	scaffold00001	Base sub	2960195	2960195
GC	scaffold00001	Base sub	3253089	3253089
GC	scaffold00002	Base sub	604268	604268
GC	scaffold00002	Deletion	3336710	3336718
GC	scaffold00003	Base sub	2598716	2598716
GC	scaffold00003	Base sub	2616915	2616915
GC	scaffold00003	Base sub	466390	466390
GC	scaffold00004	Base sub	1944869	1944869
GC	scaffold00004	Base sub	2156727	2156727
GC	scaffold00004	Base sub	610899	610899
GC	scaffold00004	Base sub	610900	610900
GC	scaffold00004	Insertion	2142114	2142114
GC	scaffold00005	Base sub	2356972	2356972
GC	scaffold00005	Base sub	3390733	3390733
GC	scaffold00005	Base sub	348927	348927
GC	scaffold00005	Insertion	978421	978421
GC	scaffold00006	Base sub	1795707	1795707
GC	scaffold00007	Base sub	2431935	2431935
GC	scaffold00007	Base sub	2602372	2602372
GC	scaffold00008	Base sub	664667	664667

GC	scaffold00009	Base sub	1533409	1533409
GC	scaffold00010	Base sub	1325431	1325431
GC	scaffold00010	Insertion	1438296	1438296
GC	scaffold00011	Base sub	1204421	1204421
GC	scaffold00011	Base sub	1600208	1600208
GC	scaffold00012	Base sub	1474963	1474963
GC	scaffold00013	Insertion	2389205	2389205
GC	scaffold00014	Base sub	132026	132026
GC	scaffold00014	Base sub	1985787	1985787
GC	scaffold00015	Base sub	163601	163601
GC	scaffold00015	Base sub	163623	163623
GC	scaffold00015	Base sub	562212	562212
GC	scaffold00016	Base sub	1269291	1269291
GC	scaffold00016	Base sub	526561	526561
GC	scaffold00017	Base sub	505739	505739
GC	scaffold00018	Base sub	116207	116207
GC	scaffold00020	Base sub	1148847	1148847
GC	scaffold00021	Base sub	968523	968523
GC	scaffold00024	Insertion	685658	685658
GC	scaffold00029	Base sub	340459	340459
GC	scaffold00030	Base sub	1145780	1145780
GC	scaffold00035	Base sub	316680	316680
GC	scaffold00035	Base sub	774890	774890
GC	scaffold00038	Base sub	365463	365463
GC	scaffold00049	Base sub	141927	141927
GC	scaffold00056	Base sub	373638	373638
GC	scaffold00061	Base sub	475490	475490
GC	scaffold00062	Base sub	70908	70908
GC	scaffold00066	Base sub	28315	28315
GC	scaffold00076	Deletion	219022	219032
GC	scaffold00148	Deletion	8458	8462
GC	scaffold04154	Base sub	16535	16535
GC	scaffold04746	Base sub	5034	5034
GC	scaffold07343	Base sub	114	114
GC	scaffold07901	Base sub	3024	3024
GC	scaffold08152	Base sub	2702	2702
GC	scaffold11325	Base sub	2079	2079
GC	scaffold12420	Base sub	13049	13049
GC	scaffold12574	Deletion	20748	20750
GC	scaffold14616	Base sub	4668	4668
GC	scaffold15455	Base sub	240	240

GC	scaffold16122	Base sub	1107	1107
IA	scaffold00001	Base sub	1011262	1011262
IA	scaffold00001	Base sub	1160253	1160253
IA	scaffold00001	Base sub	1759613	1759613
IA	scaffold00001	Base sub	3839740	3839740
IA	scaffold00001	Deletion	1289362	1289365
IA	scaffold00001	Deletion	3956373	3956375
IA	scaffold00001	Insertion	5673998	5673998
IA	scaffold00002	Base sub	147602	147602
IA	scaffold00002	Insertion	629793	629793
IA	scaffold00003	Base sub	1032479	1032479
IA	scaffold00003	Base sub	1167758	1167758
IA	scaffold00003	Base sub	712471	712471
IA	scaffold00004	Base sub	690813	690813
IA	scaffold00004	Base sub	98033	98033
IA	scaffold00005	Base sub	146515	146515
IA	scaffold00005	Base sub	2576881	2576881
IA	scaffold00005	Base sub	3068762	3068762
IA	scaffold00005	Base sub	3641741	3641741
IA	scaffold00009	Base sub	2377146	2377146
IA	scaffold00009	Base sub	2397216	2397216
IA	scaffold00009	Base sub	688667	688667
IA	scaffold00009	Insertion	1677721	1677721
IA	scaffold00010	Base sub	2586876	2586876
IA	scaffold00011	Base sub	2062821	2062821
IA	scaffold00011	Base sub	881063	881063
IA	scaffold00012	Base sub	44267	44267
IA	scaffold00014	Base sub	1616450	1616450
IA	scaffold00015	Deletion	7378	7382
IA	scaffold00016	Base sub	40084	40084
IA	scaffold00016	Deletion	865747	865778
IA	scaffold00017	Base sub	1100876	1100876
IA	scaffold00017	Base sub	615663	615663
IA	scaffold00018	Base sub	626936	626936
IA	scaffold00020	Base sub	1402250	1402250
IA	scaffold00023	Base sub	273934	273934
IA	scaffold00023	Base sub	702705	702705
IA	scaffold00023	Base sub	824350	824350
IA	scaffold00028	Base sub	480838	480838
IA	scaffold00035	Base sub	662048	662048
IA	scaffold00037	Base sub	553317	553317

IA	scaffold00037	Base sub	557909	557909
IA	scaffold00038	Base sub	505137	505137
IA	scaffold00038	Deletion	7059	7066
IA	scaffold00047	Base sub	474477	474477
IA	scaffold00066	Base sub	230873	230873
IA	scaffold00072	Base sub	59785	59785
IA	scaffold00079	Base sub	158379	158379
IA	scaffold00093	Deletion	98371	98372
IA	scaffold00110	Base sub	46390	46390
IA	scaffold01708	Base sub	3623	3623
IA	scaffold01759	Base sub	36518	36518
IA	scaffold02030	Base sub	37282	37282
IA	scaffold02358	Base sub	672	672
IA	scaffold04190	Base sub	8971	8971
IA	scaffold04790	Base sub	60485	60485
IA	scaffold05927	Base sub	7705	7705
IB	scaffold00001	Base sub	485165	485165
IB	scaffold00002	Base sub	3742880	3742880
IB	scaffold00003	Base sub	1098983	1098983
IB	scaffold00003	Base sub	144012	144012
IB	scaffold00003	Base sub	919434	919434
IB	scaffold00003	Insertion	114399	114399
IB	scaffold00006	Deletion	822916	822924
IB	scaffold00009	Base sub	1591792	1591792
IB	scaffold00009	Base sub	2031438	2031438
IB	scaffold00009	Base sub	2232906	2232906
IB	scaffold00009	Base sub	2277140	2277140
IB	scaffold00009	Insertion	330389	330389
IB	scaffold00010	Base sub	303934	303934
IB	scaffold00012	Base sub	1990739	1990739
IB	scaffold00015	Base sub	1464061	1464061
IB	scaffold00017	Base sub	1321582	1321582
IB	scaffold00017	Base sub	1350564	1350564
IB	scaffold00017	Deletion	617741	617748
IB	scaffold00021	Base sub	354697	354697
IB	scaffold00024	Base sub	1222868	1222868
IB	scaffold00024	Base sub	505363	505363
IB	scaffold00025	Base sub	55051	55051
IB	scaffold00027	Base sub	663226	663226
IB	scaffold00030	Base sub	632956	632956
IB	scaffold00033	Deletion	183933	183936

IB	scaffold00035	Base sub	461937	461937
IB	scaffold00036	Base sub	583741	583741
IB	scaffold00040	Base sub	465833	465833
IB	scaffold00042	Base sub	414356	414356
IB	scaffold00043	Base sub	549337	549337
IB	scaffold00043	Base sub	549338	549338
IB	scaffold00045	Base sub	559999	559999
IB	scaffold00045	Deletion	225769	225771
IB	scaffold00050	Base sub	30429	30429
IB	scaffold00316	Base sub	8006	8006
IB	scaffold02437	Base sub	7746	7746
IB	scaffold04408	Base sub	18703	18703
IB	scaffold04656	Insertion	36905	36905
IB	scaffold10067	Deletion	90	91
IC	scaffold00001	Base sub	2659514	2659514
IC	scaffold00001	Base sub	522752	522752
IC	scaffold00002	Base sub	1979901	1979901
IC	scaffold00002	Base sub	2917188	2917188
IC	scaffold00002	Base sub	3488187	3488187
IC	scaffold00002	Base sub	672623	672623
IC	scaffold00002	Base sub	692033	692033
IC	scaffold00002	Base sub	81170	81170
IC	scaffold00002	Base sub	954135	954135
IC	scaffold00005	Base sub	2241726	2241726
IC	scaffold00005	Base sub	532535	532535
IC	scaffold00005	Base sub	694606	694606
IC	scaffold00007	Base sub	2084698	2084698
IC	scaffold00009	Base sub	2294746	2294746
IC	scaffold00009	Deletion	2295788	2295789
IC	scaffold00010	Base sub	2219045	2219045
IC	scaffold00010	Base sub	2219076	2219076
IC	scaffold00010	Base sub	2219086	2219086
IC	scaffold00010	Insertion	1763326	1763326
IC	scaffold00011	Base sub	274458	274458
IC	scaffold00011	Base sub	547908	547908
IC	scaffold00015	Base sub	35884	35884
IC	scaffold00016	Base sub	275046	275046
IC	scaffold00017	Base sub	1382176	1382176
IC	scaffold00017	Base sub	952139	952139
IC	scaffold00020	Base sub	127910	127910
IC	scaffold00020	Base sub	354618	354618

IC	scaffold00022	Base sub	1024847	1024847
IC	scaffold00022	Base sub	1068951	1068951
IC	scaffold00022	Base sub	656768	656768
IC	scaffold00023	Base sub	1602036	1602036
IC	scaffold00023	Base sub	91724	91724
IC	scaffold00024	Base sub	710286	710286
IC	scaffold00030	Base sub	978469	978469
IC	scaffold00032	Base sub	362317	362317
IC	scaffold00034	Deletion	543669	543670
IC	scaffold00038	Base sub	112725	112725
IC	scaffold00039	Base sub	635919	635919
IC	scaffold00042	Base sub	121607	121607
IC	scaffold00042	Base sub	121608	121608
IC	scaffold00044	Insertion	183759	183759
IC	scaffold00050	Base sub	405391	405391
IC	scaffold00053	Base sub	451536	451536
IC	scaffold00060	Base sub	113697	113697
IC	scaffold01192	Base sub	8706	8706
IC	scaffold01417	Base sub	16796	16796
IC	scaffold02079	Deletion	153610	153623
IC	scaffold03560	Base sub	7083	7083
IC	scaffold03855	Base sub	21506	21506
IC	scaffold04328	Base sub	7006	7006
IC	scaffold04328	Base sub	7007	7007
IC	scaffold04749	Base sub	18384	18384
IC	scaffold06580	Base sub	5738	5738
IC	scaffold07508	Deletion	84678	84679
IC	scaffold08660	Base sub	21193	21193

Supplemental Figures

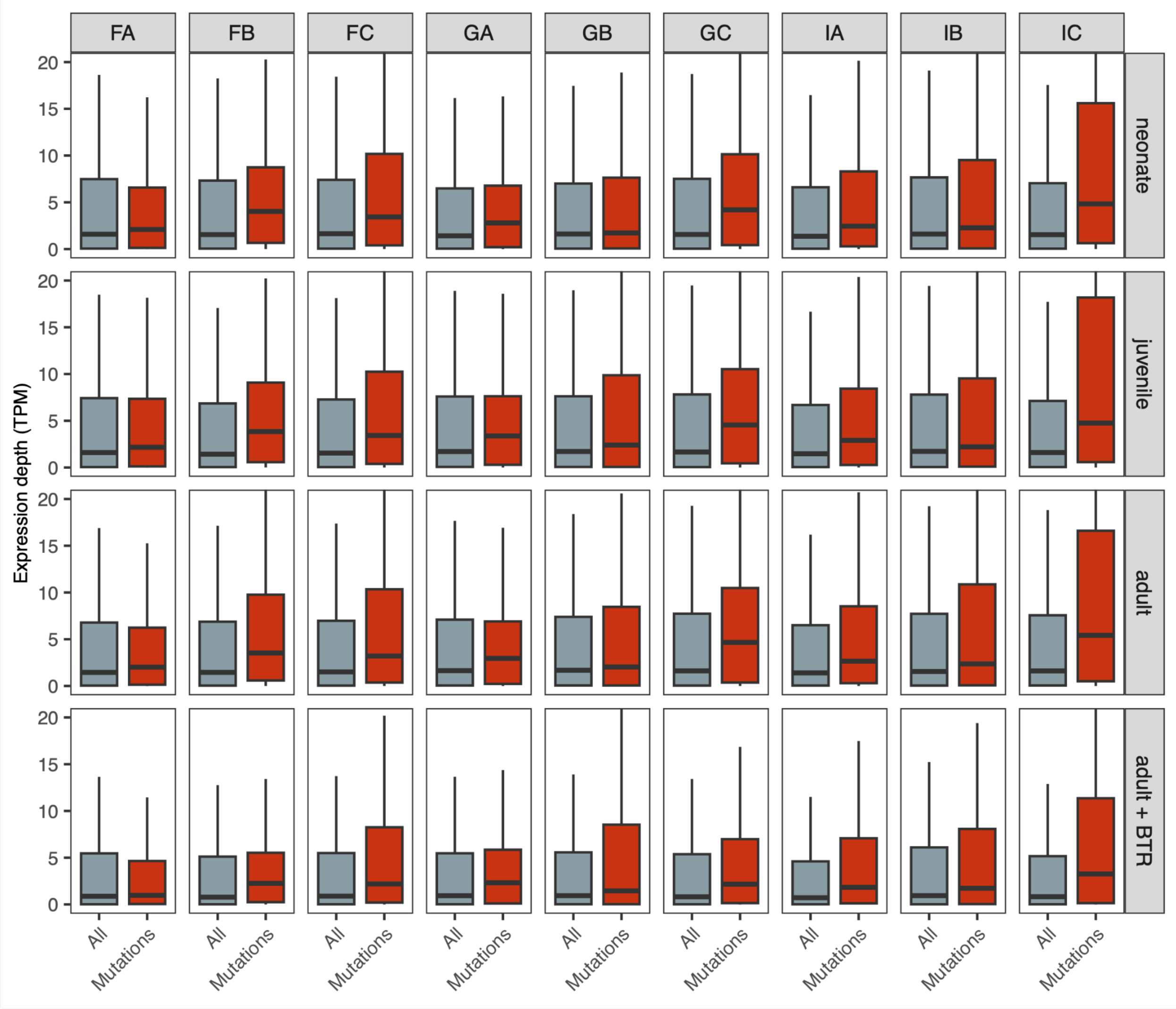

Figure S1. Median expression depth in all windows (grey) compared to windows containing mutations (red) for all combinations of genotype (top) and stage/condition (right) in *D. magna*. Median expression depth shown in transcripts per million [TPM] using 10Kb windows.

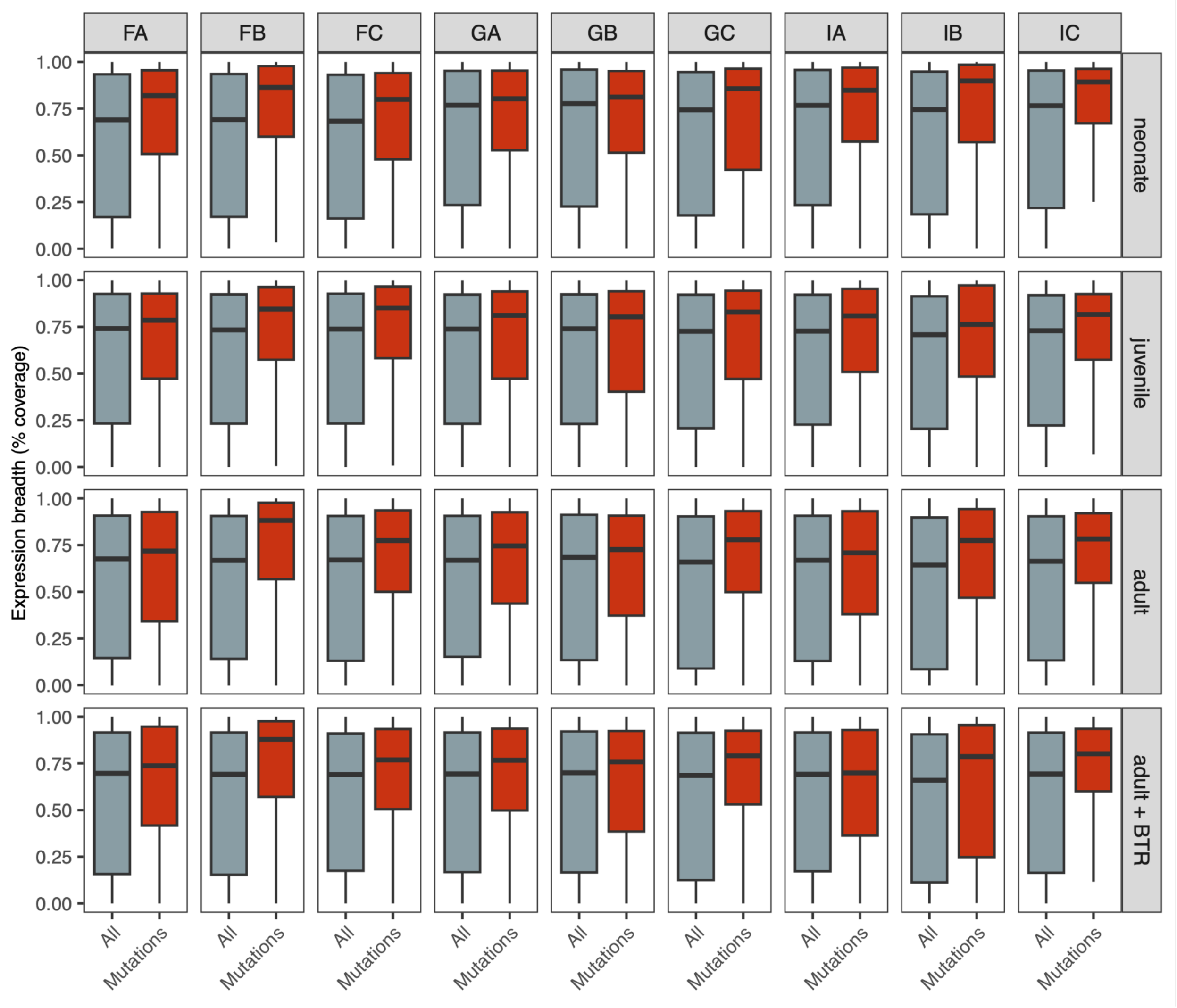

Figure S2. Median expression breadth (fraction of bases expressed) in windows with mutations (red) compared to all windows (grey), for all combinations of genotype (top) and stage/condition (right) in *D. magna* using 10Kb windows.

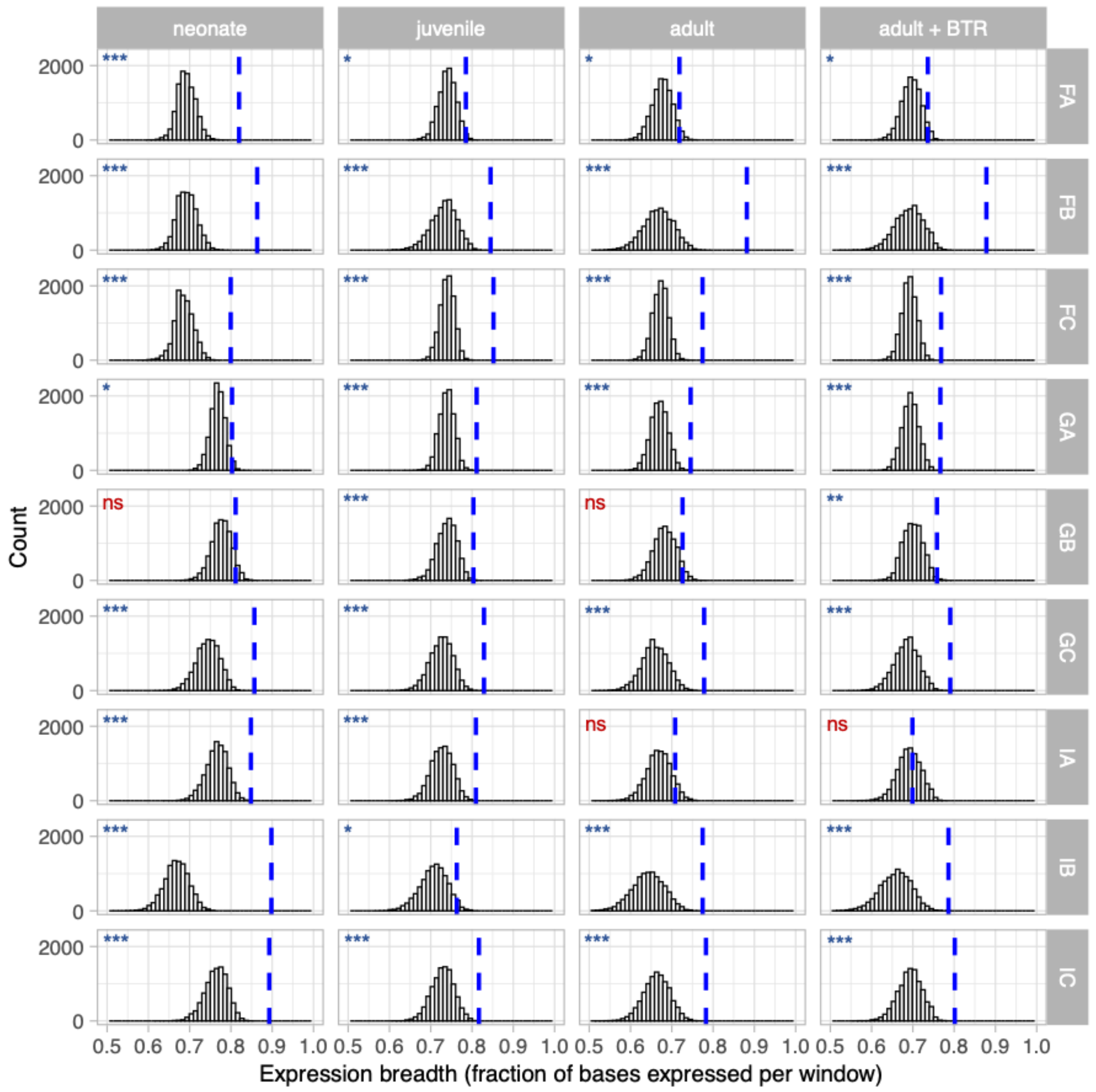

Figure S3. Permutation tests of elevated expression breadth in mutation-overlapping windows for nine genotypes, three developmental stages, and two environmental conditions (adults grown with or without exposure to BTR) in *D. magna*. Distribution of median expression depths (transcripts per million; TPM) from 10,000 permutations of randomly selected windows (bars), with the median expression breadth for mutation-containing windows (dashed blue line). Regions of the genome that were masked prior to mutation calling (see Methods) were also masked prior to calculating expression depth. Significance values are shown for each genotype-developmental stage-condition combination (*, p<0.05; **, p<0.01; ***, p<0.001; ns, p>0.05), with non-significant tests (p>0.05) shown in red.

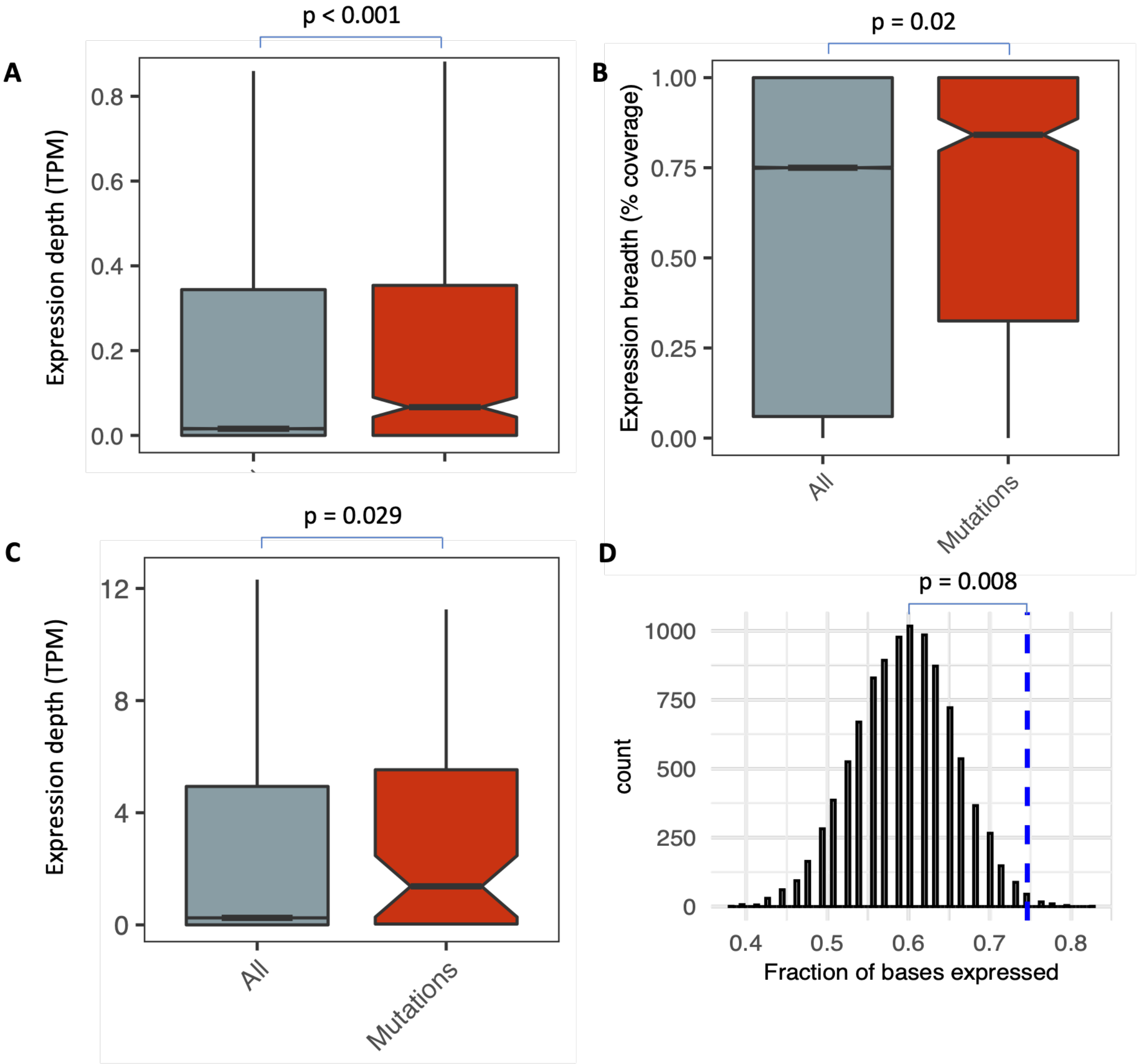

Figure S4. Expression levels (expression depth - panels A and C, and breadth - panels B and D) across all intervals in the full genome (All) vs. mutation-overlapping intervals (Mutations) in adults of a single genotype [GC] of *D. magna*. A and B, Sliding 1Kb windows (100 bp increments); C and D, Individual base pairs. For individual base pairs, because coverage is binary (covered or not, the plot shows the fraction of mutated bases that are expressed (dashed blue line) and the distribution of the equivalent fraction from 10,000 permutations of bases randomly selected from the genome (panel B, right).

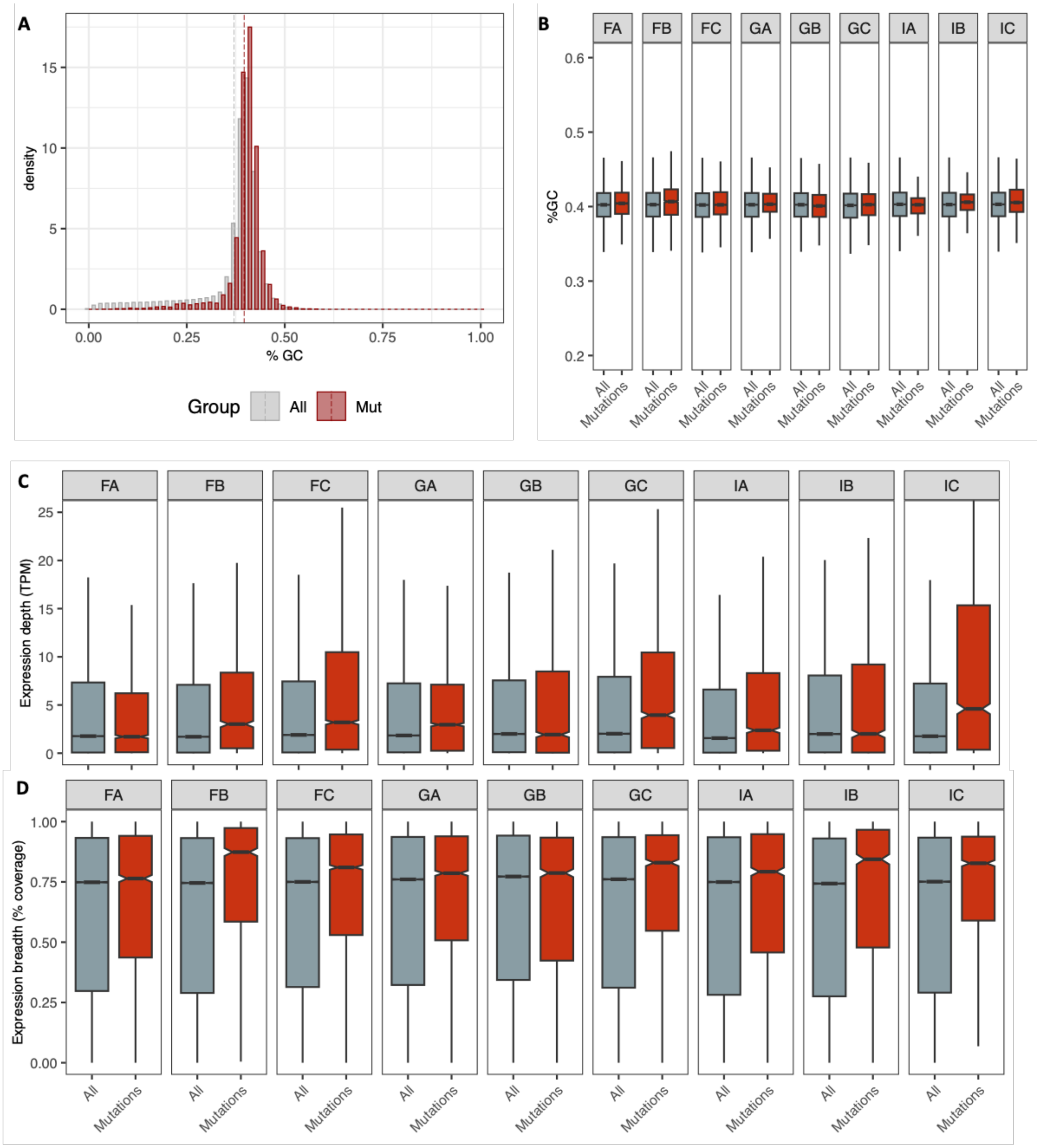

Figure S5. Expression level is elevated in mutation-overlapping windows even after controlling for GC content. A) GC content differs between all windows (grey) and windows containing mutations (red) (p<0.0001; Welch's t test), with a skew towards low-GC content (<30%) in all windows (N = 948,016 windows across 9 genotypes) relative to the subset of mutation-containing windows (N = 6,008). B) After removing windows with extreme GC contents (<30% or >50%), levels are comparable between all windows and windows containing mutations. C) Expression depth after removing low- (<30%) and high-GC (>50%) windows. D, Expression breadth after removing low- (<30%) and high-GC (>50%) windows. All plots based on 10 Kb windows.

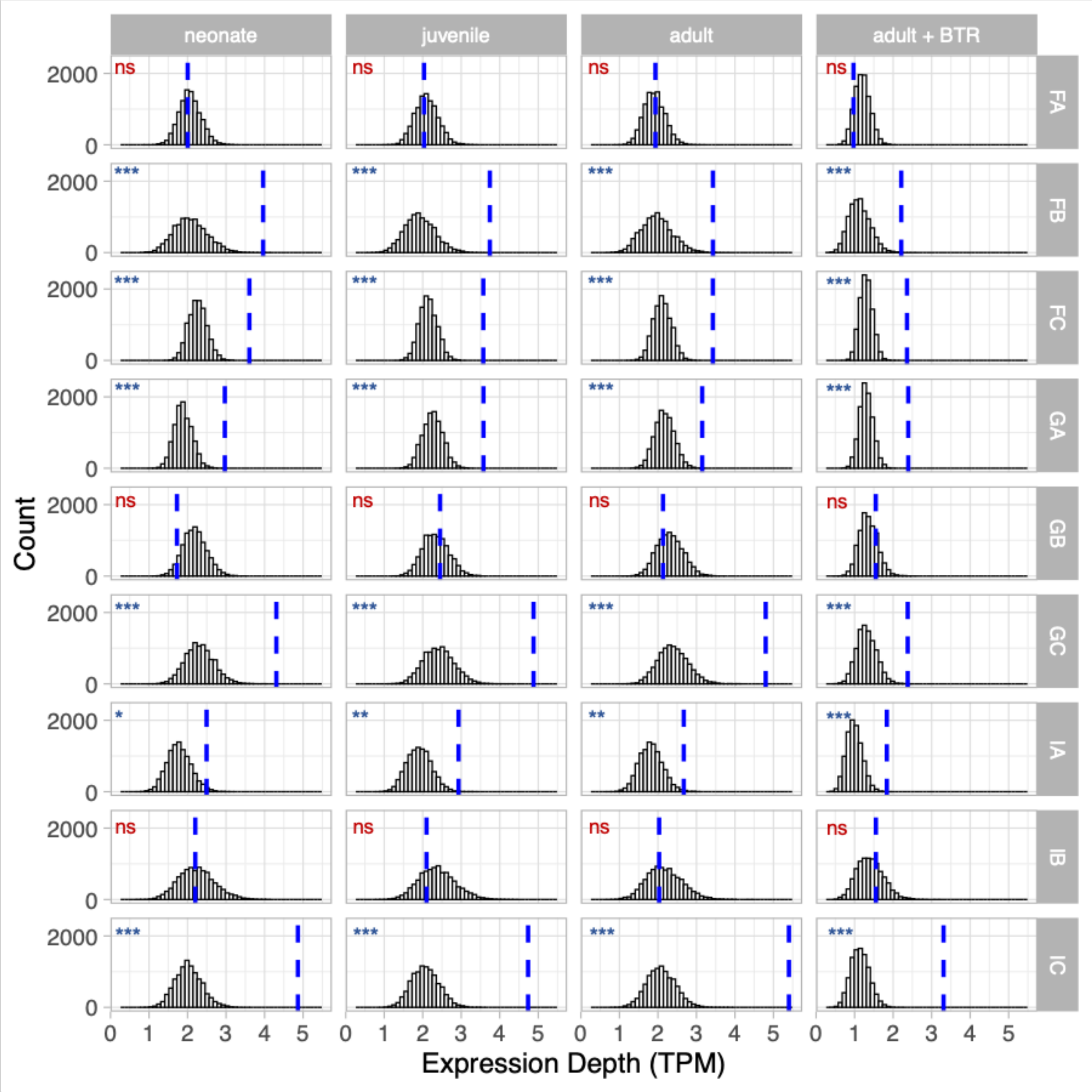

Figure S6. Permutation tests of elevated expression depth in mutation-overlapping windows for 9 genotypes, three developmental stages, and two environmental conditions (adults grown with or without exposure to BTR) in *D. magna* after excluding windows with GC content <30% or >50%. Distribution of median expression depths (transcripts per million; TPM) from 10,000 permutations of randomly selected windows (bars), with the median expression depth for mutation-containing windows (dashed blue line). Significance values are shown (*, p<0.05; **, p<0.01; ***, p<0.001; ns, p>0.05)., with non-significant tests (p>0.05) shown in red.

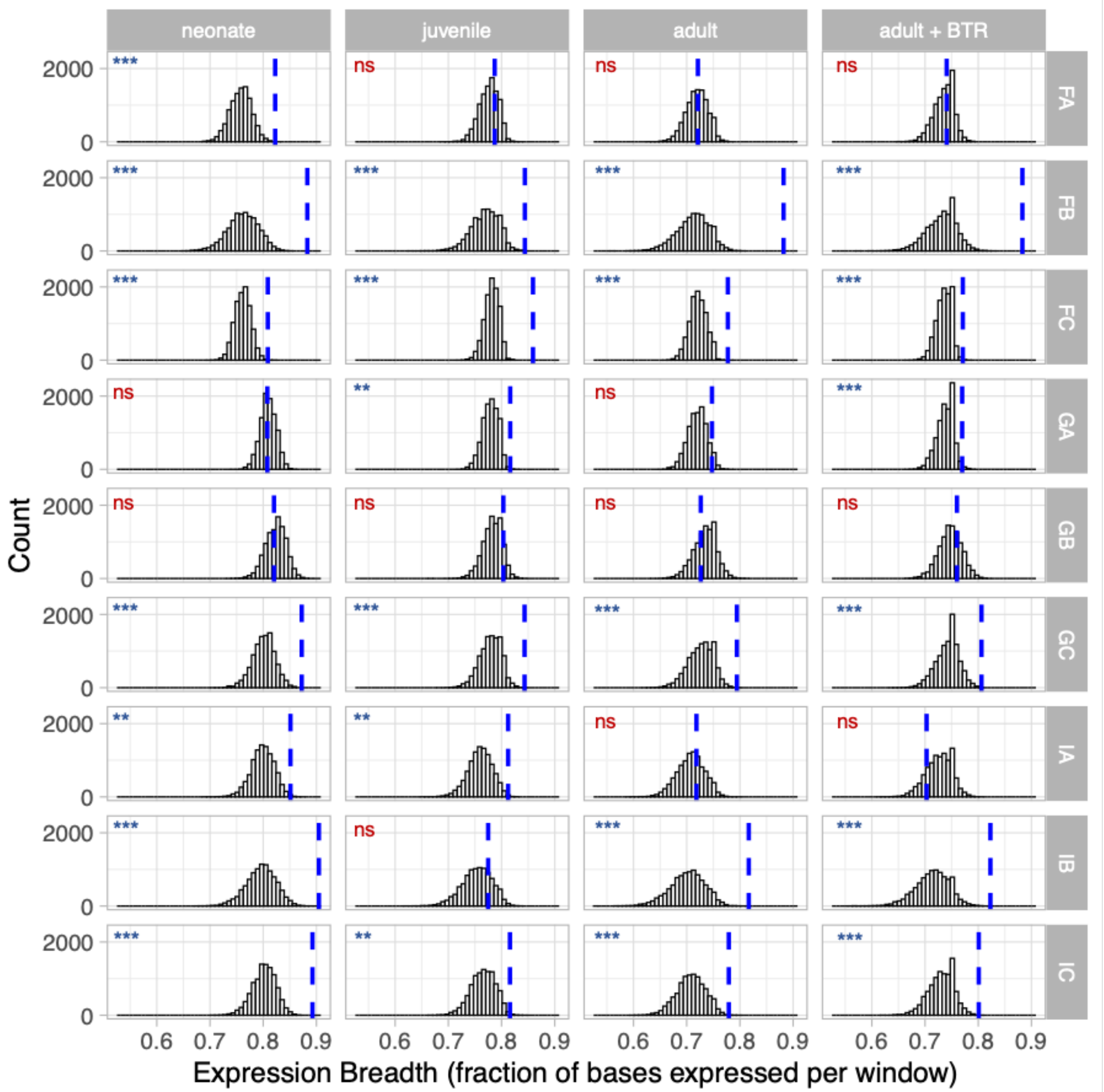

Figure S7. Permutation tests of elevated expression breadth in mutation-overlapping windows for 9 genotypes, three developmental stages, and two environmental conditions (adults grown with or without exposure to BTR) in *D. magna* after excluding windows with GC content <30% or >50%. Distribution of median expression breadths (fraction of bases expressed) from 10,000 permutations of randomly selected windows (bars), with the median expression breadth for mutation-containing windows (dashed blue line). Significance values are shown (*, p<0.05; **, p<0.01; ***, p<0.001; ns, p>0.05)., with non-significant tests (p>0.05) shown in red.

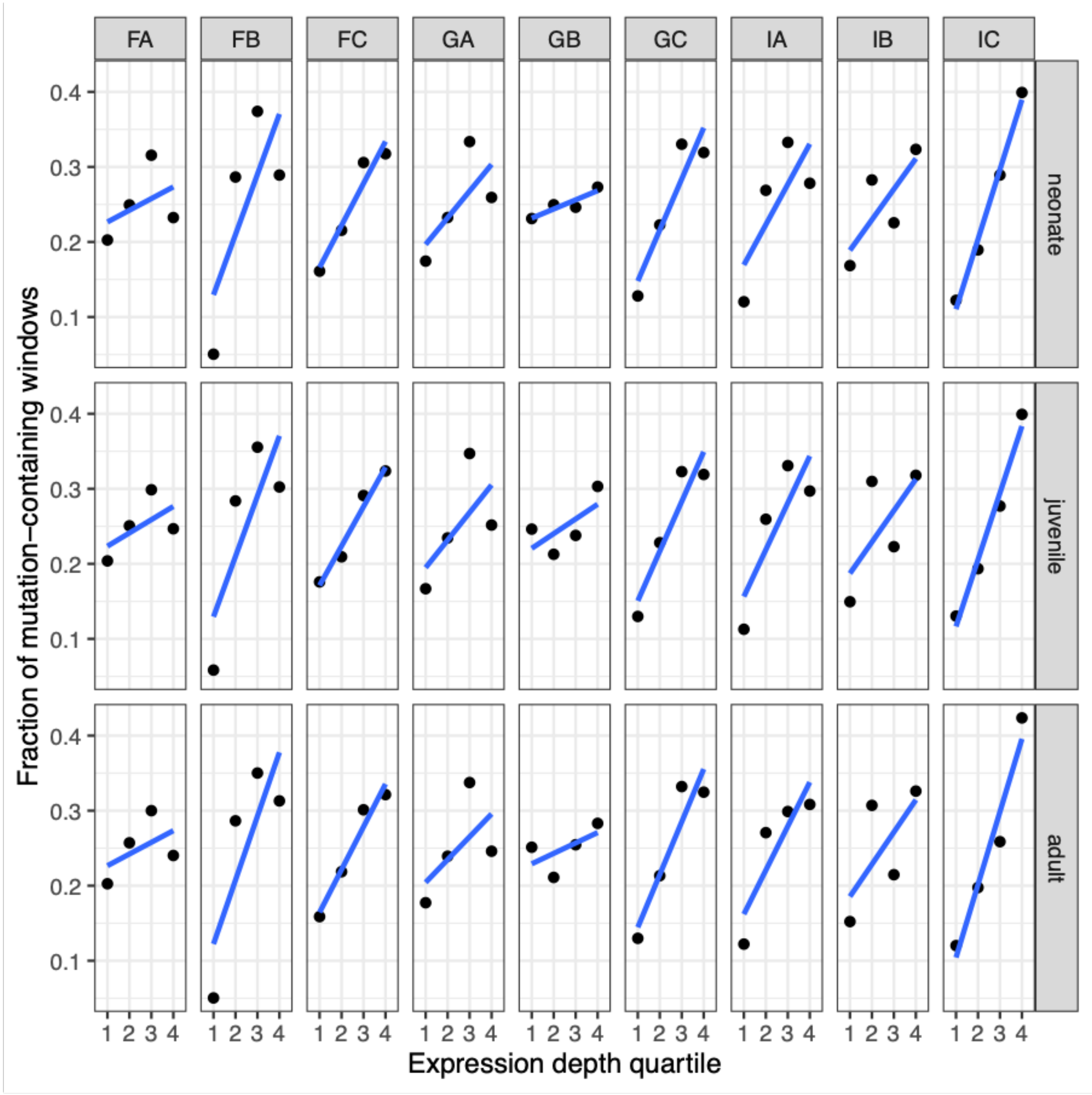

Figure S8. Low expression regions of the genome (lowest 2 quartiles) have fewer mutations than high expression regions of the genome (highest 2 quartiles). The fraction of mutation-containing windows in each expression depth quartile. On the x-axis, Quartile 1 (left) corresponds to the 25% of windows with the lowest expression depth, and Quartile 4 (right) corresponds to the 25% of windows with the highest expression depth.

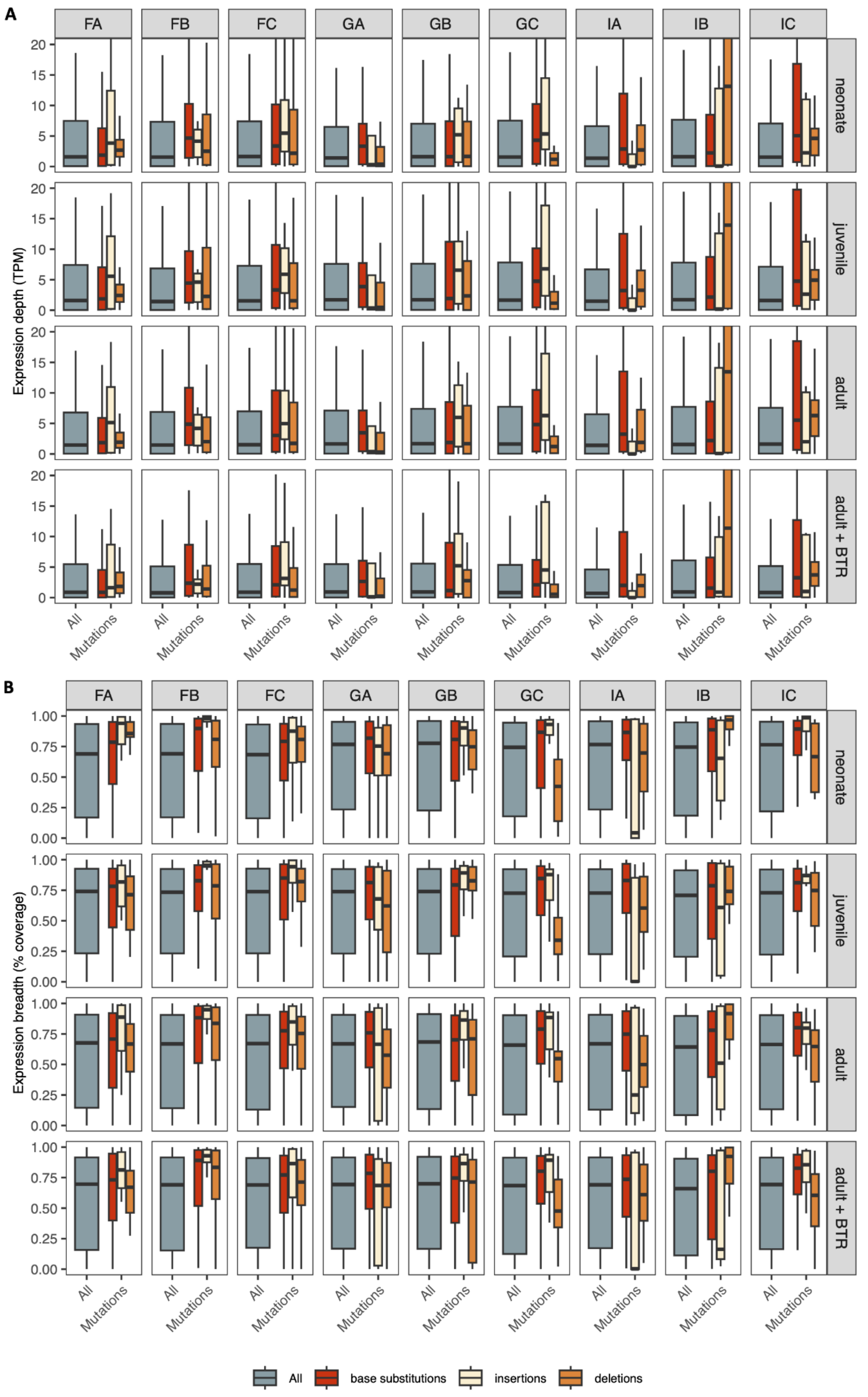
A
FA
FB
FC
GA
GB
GC
IA
IB
IC
neonate
juvenile
adult
adult + BTR
Expression depth (TPM)
All
Mutations
B
Expression breadth (% coverage)
All
base substitutions
insertions
deletions

Figure S9. The positive relationship between expression and mutation is generally observed across different mutation types (base substitutions, insertions, and deletions). Expression levels for the full genome (All; grey) and for windows overlapping mutations (Mutations; red/cream/orange), separating mutations by type (base substitution [red], insertion [cream], deletion [orange]) for each of 9 genotypes (FA-FC, GA-GC, IA-IC), three developmental stages (neonate, juvenile, adult), and one condition (adult + BTR). All plots based on 10 Kb windows.

Supplemental References

Altschul SF, Gish W, Miller W, Myers EW, Lipman DJ. 1990. Basic local alignment search tool. Journal of Molecular Biology. 215:403–410. doi: 10.1016/S0022-2836(05)80360-2.

Bankevich A et al. 2012. SPAdes: A New Genome Assembly Algorithm and Its Applications to Single-Cell Sequencing. Journal of Computational Biology. 19:455–477. doi: 10.1089/cmb.2012.0021.

Bolger AM, Lohse M, Usadel B. 2014. Trimmomatic: a flexible trimmer for Illumina sequence data. Bioinformatics. 30:2114–2120. doi: 10.1093/bioinformatics/btu170.

Bushnell B, Rood J, Singer E. 2017. BBMerge – Accurate paired shotgun read merging via overlap. PLOS ONE. 12:e0185056. doi: 10.1371/journal.pone.0185056.

Cantarel BL et al. 2008. MAKER: An easy-to-use annotation pipeline designed for emerging model organism genomes. Genome Res. 18:188–196. doi: 10.1101/gr.6743907.

Colbourne JK et al. 2011. The Ecoresponsive Genome of *Daphnia pulex*. Science. 331:555–561. doi: 10.1126/science.1197761.

Giraudo M, Douville M, Cottin G, Houde M. 2017. Transcriptomic, cellular and life-history responses of Daphnia magna chronically exposed to benzotriazoles: Endocrine-disrupting potential and molting effects. PLOS ONE. 12:e0171763. doi: 10.1371/journal.pone.0171763.

Ho EKH et al. 2020. High and Highly Variable Spontaneous Mutation Rates in Daphnia. Molecular Biology and Evolution. 37:3258–3266. doi: 10.1093/molbev/msaa142.

Ho EKH et al. 2019. Intraspecific Variation in Microsatellite Mutation Profiles in Daphnia magna Rogers, R, editor. Molecular Biology and Evolution. 36:1942–1954. doi: 10.1093/molbev/msz118.

Ho EKH, Schaack S. 2021. Intraspecific Variation in the Rates of Mutations Causing Structural Variation in Daphnia magna. Genome Biology and Evolution. 13:evab241. doi: 10.1093/gbe/evab241.

Kim D, Paggi JM, Park C, Bennett C, Salzberg SL. 2019. Graph-based genome alignment and genotyping with HISAT2 and HISAT-genotype. Nat Biotechnol. 37:907–915. doi: 10.1038/s41587-019-0201-4.

Klüttgen B, Dülmer U, Engels M, Ratte HT. 1994. ADaM, an artificial freshwater for the culture of zooplankton. Water Research. 28:743–746. doi: 10.1016/0043-1354(94)90157-0.

Korf I. 2004. Gene finding in novel genomes. BMC Bioinformatics. 5:59. doi: 10.1186/1471-2105-5-59.

Li H, Durbin R. 2010. Fast and accurate long-read alignment with Burrows–Wheeler transform. Bioinformatics. 26:589–595. doi: 10.1093/bioinformatics/btp698.

Poulsen R, De Fine Licht HH, Hansen M, Cedergreen N. 2021. Grandmother's pesticide exposure revealed bi-generational effects in Daphnia magna. Aquatic Toxicology. 236:105861. doi: 10.1016/j.aquatox.2021.105861.

Pryszcz LP, Gabaldón T. 2016. Redundans: an assembly pipeline for highly heterozygous genomes. Nucleic Acids Research. 44:e113. doi: 10.1093/nar/gkw294.

Quinlan AR, Hall IM. 2010. BEDTools: a flexible suite of utilities for comparing genomic features. Bioinformatics. 26:841–842. doi: 10.1093/bioinformatics/btq033.

Russo C, Isidori M, Deaver JA, Poynton HC. 2018. Toxicogenomic responses of low level anticancer drug exposures in *Daphnia magna*. Aquatic Toxicology. 203:40–50. doi: 10.1016/j.aquatox.2018.07.010.

Smit A, Hubley R. 2008. RepeatModeler Open-1.0. <http://www.repeatmasker.org>.

Smit A, Hubley R, Green P. 2013. RepeatMasker Open-4.0. http://www.repeatmasker.org.

Stanke M et al. 2006. AUGUSTUS: ab initio prediction of alternative transcripts. Nucleic Acids Research. 34:W435–W439. doi: 10.1093/nar/gkl200.

Timothy J. Boerner, Stephen Deems, Thomas R. Furlani, Shelley L. Knuth, and John Towns. 2023. ACCESS: Advancing Innovation: NSF's Advanced Cyberinfrastructure Coordination Ecosystem: Services & Support. "In Practice and Experience in Advanced

Research Computing (PEARC ’23)”, July 23–27, 2023, Portland, OR, USA. ACM, New York, NY, USA, 4 pages. https://doi.org/10.1145/3569951.3597559.

Toyota K et al. 2015. NMDA receptor activation upstream of methyl farnesoate signaling for short day-induced male offspring production in the water flea, Daphnia pulex. BMC Genomics. 16:186. doi: 10.1186/s12864-015-1392-9.

Appendix

R scripts to perform permutation tests.

Scripts are shown for performing the permutation test on the *D. pulex* ovary dataset where “allmeans” is an R dataframe in which each row is a 10 kb region (“window”) of the *D. pulex* genome, and columns are as follows:

1. Type (“All” = all windows across the genome; “Mut” = a window that contains a mutation)
2. Group (For windows of Type “Mut” - what type of mutation is present [insertion, deletion, SNM])
3. Scaffold (scaffold ID from the genome assembly)
4. Start_coord (start coordinate on the scaffold)
5. Pct_cov (percent coverage; percentage of bases in the window covered by at least one RNA-seq read, aka, “expression breadth”)
6. Count (number of reads overlapping the window)
7. TPM (Transcripts per Million; aka “expression depth”)

—--

```
 #subset full dataset to mutation-containing windows
  pulexmut<-subset(allmeans, Type=="Mut")

  # Set seed for reproducible results
  set.seed(92)

  # Initialize a list to store the simulated test-statistics
  simulated_count_means <- list()
  simulated_cov_means <- list()
  simulated_count_medians <- list()
  simulated_cov_medians <- list()

 # set number of iterations to 10,000
  nreps = 10000
```

```
# Iterate
 for(i in 1:nreps){

   # Create temporary dataframe to permute so we don't modify the original
   reshuffled <- allmeans

   # Permute the expression columns with the 'sample()' function.
   count <- sample(reshuffled$TPM, size = length(pulexmut$Type),
            replace = FALSE)
   cov<-sample(reshuffled$Pct_cov, size = length(pulexmut$Type),
            replace = FALSE)

   # Calculate the means and medians for each permutation
   mean_TPM <- mean(count)
   mean_cov <- mean(cov)
   med_TPM <- median(count)
   med_cov <- median(cov)

   # Append simulated mean difference to list
   simulated_count_means[i] <- mean_TPM
   simulated_cov_means[i] <- mean_cov
   simulated_count_medians[i] <- med_TPM
   simulated_cov_medians[i] <- med_cov
  }

 # Unlist simulated means list into numeric vector
 simulated_count_means <- unlist(simulated_count_means)
 simulated_cov_means <- unlist(simulated_cov_means)
 simulated_count_medians <- unlist(simulated_count_medians)
 simulated_cov_medians <- unlist(simulated_cov_medians)

 aa <-data.frame(simulated_count_means,simulated_cov_means,
            simulated_count_medians,simulated_cov_medians)

# Calculate mean and median expression values for observed data
 median <- pulexmut %>%
   summarise (med_cov=median(Pct_cov),
            med_TPM=median(TPM),
            mean_cov=mean(Pct_cov),
            mean_TPM=mean(TPM))

# Plot observed median TPM against distribution of median TPMs from permutations
 ggplot(aa, aes(x=simulated_count_medians)) +
```

```
geom_histogram(bins=100, aes(y=..count..), fill="white", color="black",
            position="dodge") +
geom_vline(data=median, aes(xintercept = med_TPM),
         size = 0.8, linetype = "dashed", colour = "blue") +
theme(axis.text.y = element_text(size=8)) +
theme_minimal() +
xlab("Expression (TPM)")
```
